# Molecular Dynamics Simulations Reveal Structural Changes Associated with the ABCA1 R230C Functional Variant

**DOI:** 10.3390/ijms27188240

**Published:** 2026-09-16

**Authors:** Juan José de la Cruz-López, Luis Ramón Tercero Martínez-González, Cecilia Albortante-Morato, Rosaura Palma-Orozco, María Teresa Flores-Dorantes, Jorge Luis Rosas-Trigueros

**Affiliations:** 1 Laboratorio de Biología Molecular y Farmacogenómica, Centro de Investigación de Ciencia y Tecnología Aplicada de Tabasco (CICTAT), División Académica de Ciencias Básicas (DACB), Universidad Juárez Autónoma de Tabasco (UJAT), Carretera Cunduacán-Jalpa de Méndez Km. 1, Cunduacán C.P. 86690, Tabasco, Mexico; juan.delacruzlopez@ujat.mx (J.J.d.l.C.-L.);; 2 División Académica de Ciencias y Tecnologías de la Información (DACYTI), Centro de Investigación de Ciencia y Tecnología Aplicada de Tabasco (CICTAT), Universidad Juárez Autónoma de Tabasco (UJAT), Carretera Cunduacán-Jalpa de Méndez Km. 1, Cunduacán C.P. 86690, Tabasco, Mexico; 3 Laboratorio Transdisciplinario de Investigación en Sistemas Evolutivos, Escuela Superior de Cómputo (ESCOM), Instituto Politécnico Nacional (IPN), Ciudad de México 07738, Mexicorpalma@ipn.mx (R.P.-O.)

**Keywords:** ABCA1 membrane protein, R230C variant, cholesterol efflux, HDL cholesterol, Protein Structure, hypoalphalipoproteinemia

## Abstract

The ATP-binding cassette transporter A1 (ABCA1) functional variant R230C (rs9282541) is associated with low plasma HDL-C levels, yet its atomic-scale mechanism remains unclear. We evaluated the structural and dynamic impact of R230C compared to wild-type (WT) ABCA1 using 200 ns molecular dynamics simulations in a lipid raft membrane. While the tertiary fold was preserved, R230C exhibited reduced overall flexibility (lower RMSD and localized RMSF rigidification) alongside a slightly expanded conformation (increased Rg and SASA). Localized fluctuations near residue 230 in extracellular domain 1 (ECD1) were coupled with decreased dynamic heterogeneity in distal functional regions, particularly nucleotide-binding domain 2 (NBD2) and transmembrane domain 2 (TMD2). MOSAICS analysis revealed subtle alterations in membrane thickness, midplane displacement, and lipid orientation. Furthermore, CAVER tunnel analysis demonstrated increased pathway heterogeneity (17 clusters in R230C vs. 10 in WT), lower persistence, and smaller bottleneck radii, disrupting the primary cholesterol transport route. Thus, R230C acts as a dynamic allosteric modulator and membrane-coupling agent rather than a folding-disruptive mutation, providing a biophysical rationale for reduced cholesterol efflux and low plasma HDL-C levels.

## 1. Introduction

The ABCA1 Cholesterol Transporter. The ATP-binding cassette transporter A1 (ABCA1) belongs to the ATP-binding cassette (ABC) superfamily, which comprises 49 transporters grouped into seven subfamilies (ABCA–ABCG) in mammals [1]. These proteins translocate various substances (lipids, sterols, metabolites, and drugs) across cell membranes. Although they are generally located in the plasma membrane, some are expressed in intracellular organelles, such as the Golgi apparatus and the endoplasmic reticulum [2,3]. Given its central role in HDL biogenesis and cholesterol efflux to apolipoprotein A-I, ABCA1 is recognized as one of the best-characterized and most extensively studied transporters involved in reverse cholesterol transport (RCT) [4,5].

The *ABCA1* gene is located on chromosome 9q31.1 and comprises 50 exons, encoding a 2261-amino acid integral membrane protein composed of two transmembrane domains (TMD1 and TMD2), each containing six transmembrane helices, two nucleotide-binding domains (NBD1 and NBD2), two large, highly glycosylated extracellular domains (ECD1 and ECD2), and two regulatory domains (RD1 and RD2) [6]. Severe mutations in this gene result in Tangier disease, an autosomal recessive disorder marked by near-total HDL-C deficiency and cholesterol ester accumulation in macrophages (foam cells) [7,8].

Cholesterol and Phospholipid Efflux Mechanism and Role of ApoA-I. According to the current model, ABCA1 generates lipid-rich membrane domains that promote membrane curvature and facilitate interaction with apolipoprotein A-I (ApoA-I) [9,10]. Multiple molecular mechanisms contribute to the regulation of ABCA1 activity, including JAK2-mediated signaling and the formation of the intramolecular disulfide bond between cysteines C75 and C309 within the first extracellular domain, both of which have been implicated in ApoA-I binding and cholesterol efflux [7,11]. Because cholesterol transport by ABCA1 depends on coordinated conformational transitions, membrane interactions, and the integrity of its internal transport pathway, alterations in protein dynamics may have important functional consequences even in the absence of major structural changes.

The *ABCA1* Gene Variants. The functional consequences of ABCA1 mutations are heterogeneous and largely determine the clinical variability observed in disorders such as Tangier disease (N875S, M1091T, C1477R, etc.) and familial hypoalphalipoproteinemia [12,13]. Studies have demonstrated that different missense variants differentially impair ABCA1 localization, apolipoprotein A-I (ApoA-I) binding, and the efflux of cholesterol and phospholipids, resulting in a broad spectrum of plasma HDL cholesterol levels and lipid phenotypes. These findings highlight that the severity and nature of ABCA1 dysfunction directly influence HDL biogenesis and disease presentation [8].

The R230C variant (rs9282541) involves a non-synonymous substitution of arginine (Arg/R) with cysteine (Cys/C) at residue 230, located in the first extracellular loop. Unlike arginine, which is basic and positively charged, cysteine contains a sulfhydryl group capable of forming disulfide bonds [14,15]. This variant is exclusive to Native American populations and has been reported at frequencies of 10–40% in indigenous and mestizo Mexican groups [16]. Clinical studies have associated this variant with hypoalphalipoproteinemia [17,18,19], obesity [17], and type 2 diabetes [20]. Functionally, it has been reported to reduce cholesterol efflux by approximately 27% [16]; however, no structural model currently explains how this amino acid substitution affects the transporter’s molecular machinery.

Molecular modeling studies of ABCA1. Recent advances in cryo-electron microscopy and molecular modeling have provided valuable insights into the nucleotide-free outward-facing conformation of ABCA1 [10,15,21]. Structural analyses and molecular dynamics simulations (MDS) have shown that disease-associated variants can perturb domain interactions, alter protein flexibility, and affect the stability of functionally relevant structural elements involved in lipid transport [14,22]. Consequently, computational approaches have become valuable tools for predicting the structural and functional consequences of ABCA1 variants, complementing genetic, biochemical, and experimental evidence. Although the functional effects of the *ABCA1* R230C variant have been demonstrated through epidemiological and experimental studies, its structural and dynamic consequences remain incompletely understood at the molecular level. Therefore, elucidating how the R230C substitution affects the conformational landscape of ABCA1 may provide a structural basis for understanding its impaired cholesterol efflux and the increased risk of metabolic disease associated with this variant.

## 2. Results

**Figure 1:**
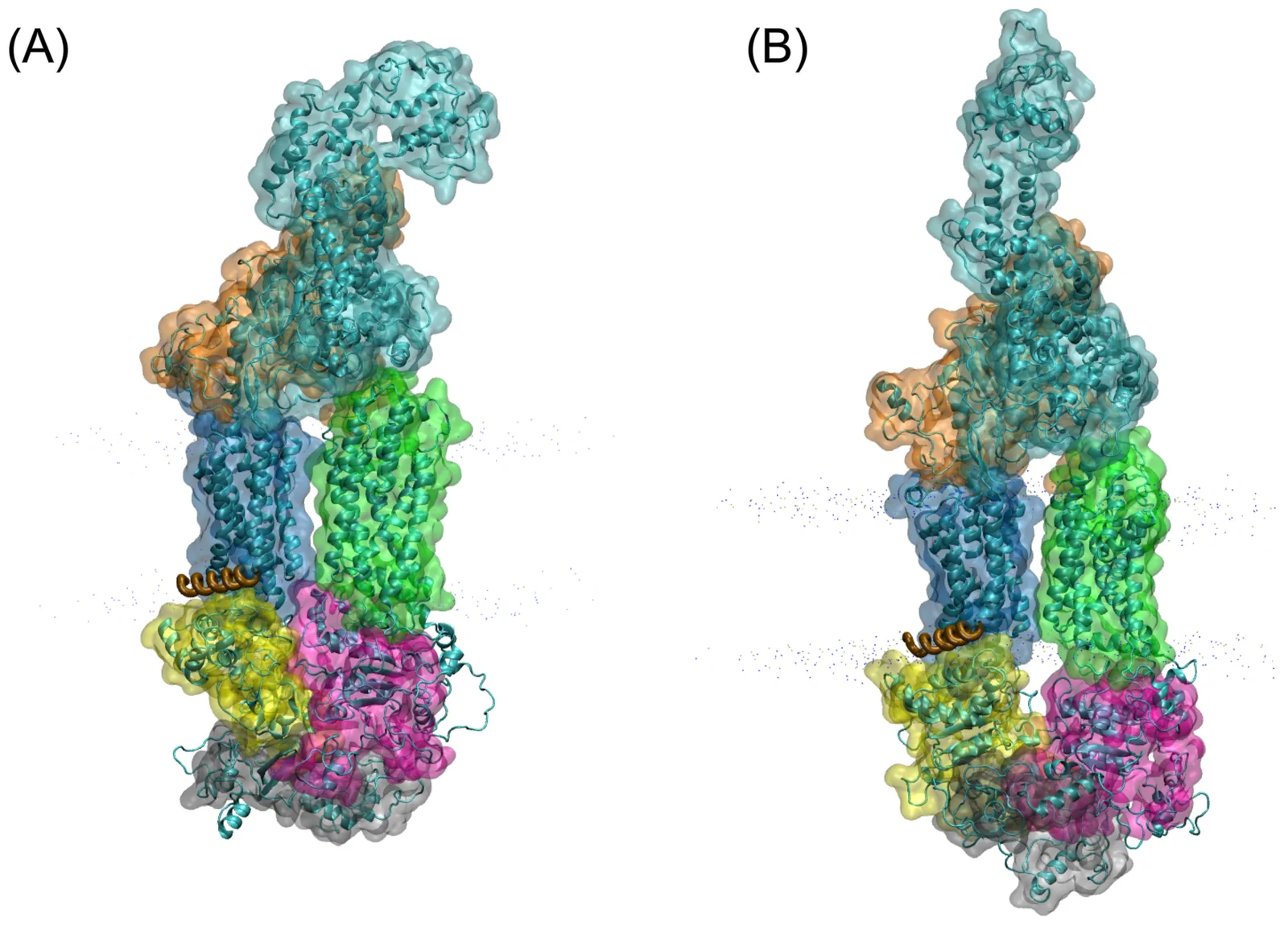
Final conformations of human ABCA1 molecular dynamics simulations. (**A**) ABCA1 WT and (**B**) the R230C variant embedded in a phospholipid bilayer. Protein domains are color-coded as follows: ECD1, teal; ECD2, orange; TMD1, blue; TMD2, green; NBD1, yellow; NBD2, pink; and RD1 and RD2, gray. The membrane boundaries are indicated by the nitrogen atoms of the lipid headgroups (blue dots).

To provide an overview of the simulated systems, the final conformations of ABCA1 WT and the R230C variant after MD simulations are shown in Figure 1. Both proteins retained the characteristic architecture of the transporter, including the large ECDs connected to two TMDs and cytoplasmic NBDs and RDs, with no evidence of global structural disruption. Although the overall fold remained conserved in both proteins, the molecular dynamics trajectories revealed subtle differences in their conformational behavior. These final conformations served as the starting point for the subsequent comparative analyses of structural stability, secondary structure, membrane interactions, transport pathway organization, and conformational dynamics.

### 2.1. MD Trajectory Analysis

To evaluate the structural stability and conformational behavior of ABCA1 during the simulations, the molecular dynamics trajectories of the WT and R230C proteins were analyzed using a series of complementary structural descriptors. Global and local protein dynamics were assessed through root mean square deviation (RMSD), root mean square fluctuation (RMSF), radius of gyration (Rg), solvent-accessible surface area (SASA), and secondary structure analyses. Together, these parameters provide a comprehensive assessment of the structural stability, flexibility, compactness, and conformational changes of both systems throughout the 200 ns simulations.

**Figure 2:**
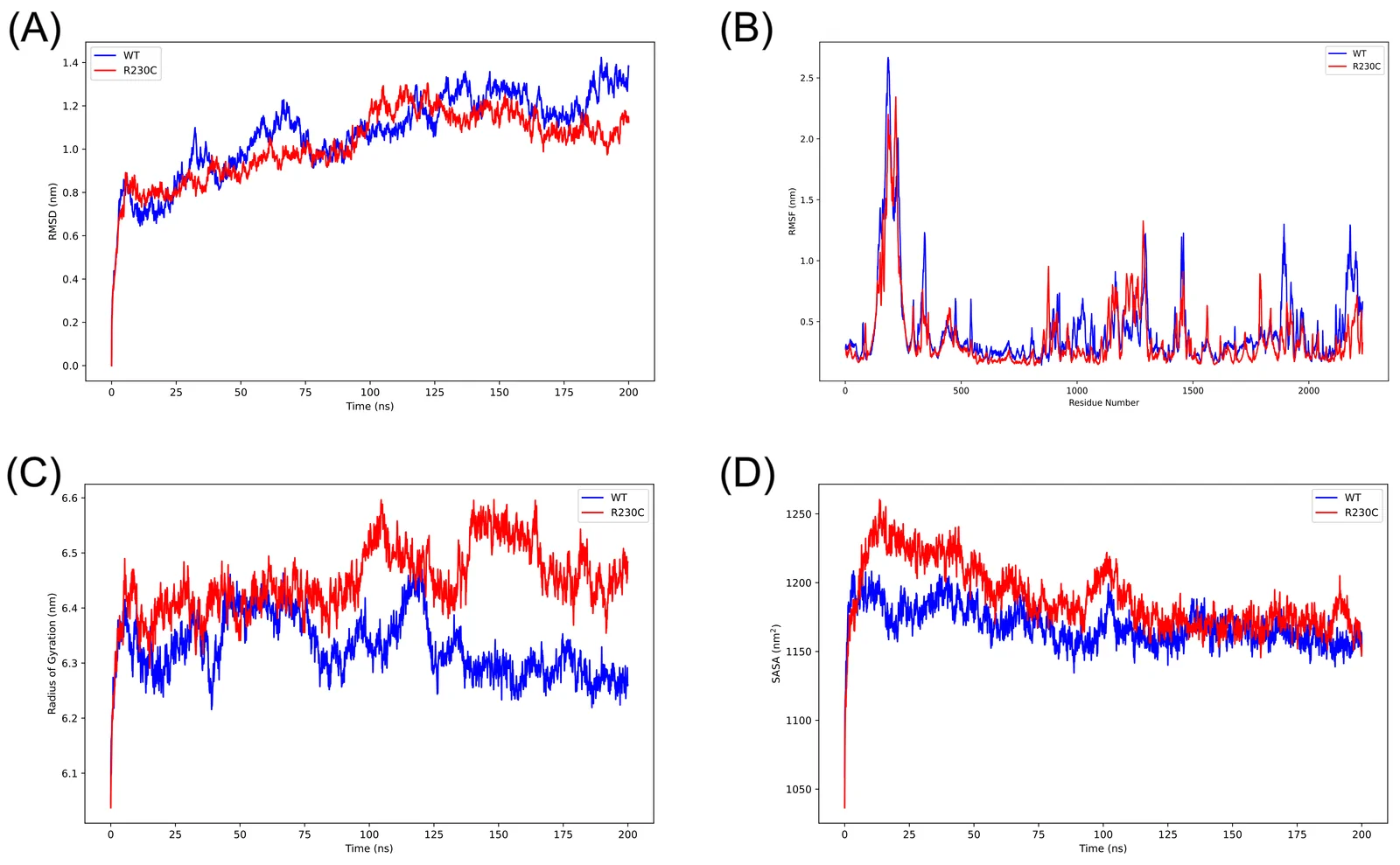
Structural Dynamics Comparison of ABCA1 Between Wild-Type (blue) and R230C variant (red): Molecular Dynamic simulation trajectories of (**A**) RMSD, (**B**) RMSF, (**C**) Rg and (**D**) SASA.

Comparison of the RMSD profiles revealed distinct conformational dynamics between ABCA1 WT and the R230C variant throughout the simulations (Figure 2A). Both proteins exhibited a rapid increase in RMSD during the initial 5 ns, indicating structural relaxation from the starting conformation. Following equilibration, comparable RMSD values were observed until approximately 90–100 ns. Thereafter, differences in their dynamic behavior became evident. The WT protein displayed a gradual increase in RMSD, reaching approximately 1.3–1.4 nm toward the end of the simulation, whereas the R230C variant exhibited lower structural deviations, fluctuating predominantly between 1.0 and 1.2 nm after 120 ns. These findings indicate that the R230C substitution alters the conformational dynamics of ABCA1 by reducing the magnitude of structural deviations during the later stages of the simulation.

The RMSF profiles were compared to evaluate the effect of the R230C substitution on residue-level flexibility throughout the MD simulations (Figure 2B). Overall, both proteins exhibited similar fluctuation patterns, indicating that the substitution did not induce large-scale changes in the dynamic behavior of ABCA1. The highest RMSF values were observed in the N-terminal region (residues 150–200), where fluctuations exceeded 2.5 nm in both proteins, although the WT protein displayed slightly greater mobility than the R230C variant, particularly around residues 300–350, 800–1200 and 1800–2200. Outside this region, most residues exhibited fluctuations of approximately 0.5 nm, with several localized peaks distributed along the sequence. In general, the R230C variant showed lower residue fluctuations than the WT protein, although moderate increases in mobility were observed at selected positions within residues 800, 1200–1300 and near residue 1800 in the R230C. The major flexibility hotspots were conserved in both trajectories, suggesting that the R230C substitution primarily restricts local residue mobility without substantially altering the global dynamic behavior of ABCA1.

The Rg profiles remained relatively stable throughout the simulation, with no abrupt changes indicative of major unfolding events (Figure 2C). The WT protein maintained values of approximately 6.30–6.40 nm, consistent with preservation of its overall compactness. In contrast, the R230C variant consistently exhibited higher Rg values, particularly after the first 20 ns, fluctuating predominantly between 6.40 and 6.60 nm. The higher values and greater fluctuation amplitude observed for the variant indicate reduced structural compactness and a moderately more expanded conformation relative to the WT protein. These results suggest that the R230C substitution influences the overall structural organization of ABCA1, resulting in a less compact conformation compared with the wild-type protein.

The SASA profiles remained relatively stable throughout the simulation for both proteins (Figure 2D). However, the R230C variant exhibited consistently higher SASA values than the WT protein during the first half of the trajectory, indicating greater solvent exposure. After approximately 120 ns, the SASA values of both systems converged and remained comparable until the end of the simulation. Overall, the higher SASA observed during the initial half of the simulation for the R230C variant suggests that the Arg230Cys substitution increases solvent accessibility, consistent with the moderately more expanded conformation indicated by the Rg analysis.

**Figure 3:**
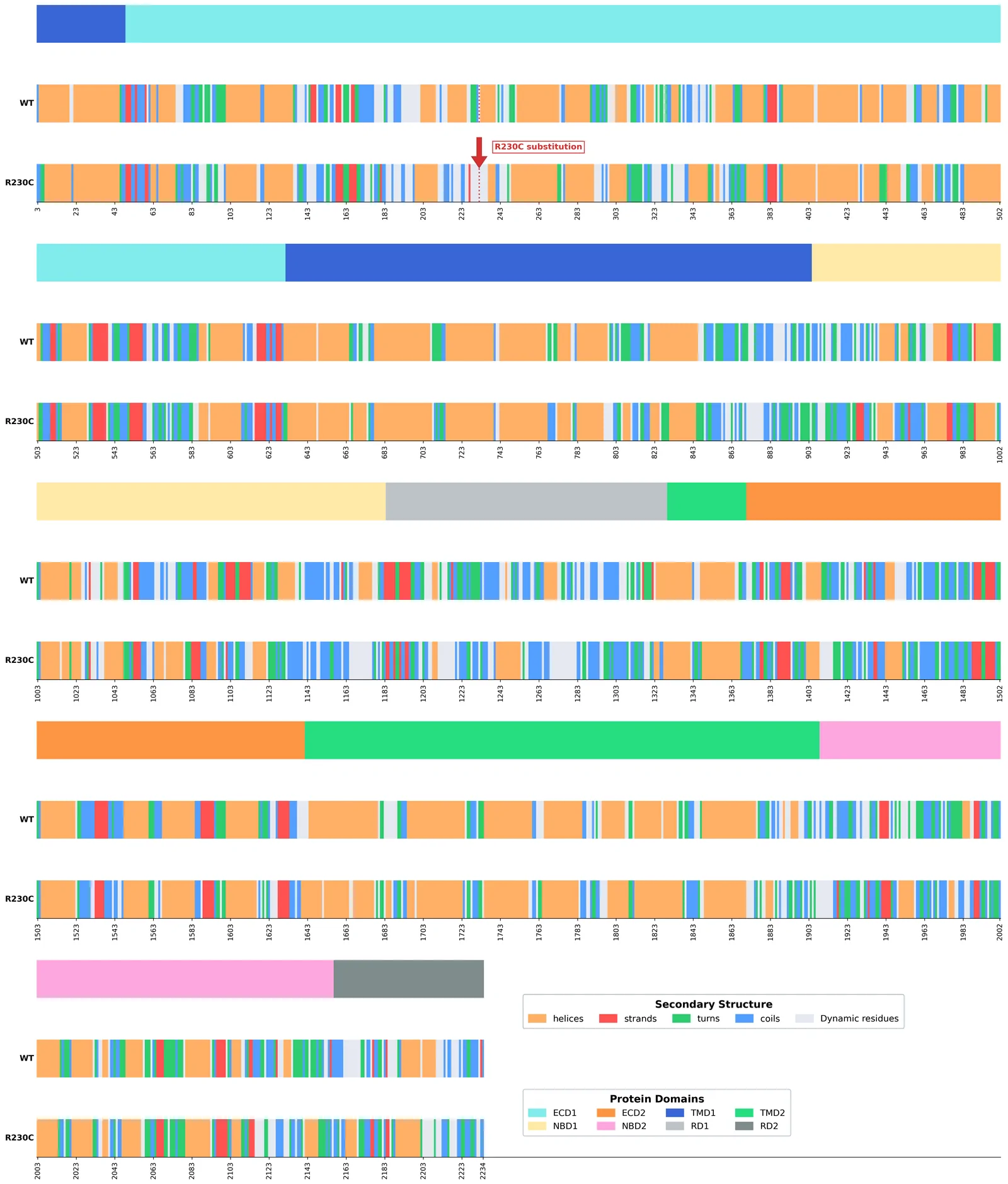
Comparative analysis of the secondary structure distribution in wild-type ABCA1 and the ABCA1 R230C variant. Secondary structure assignments obtained from MD trajectories were mapped along the ABCA1 sequence and compared between the WT protein and the R230C variant. The protein sequence was partitioned into consecutive 500-residue segments to facilitate visualization of structural features across the 2232-residue construct used in the analysis, where the final 29 residues were excluded from the simulated construct. Secondary structure elements were classified according to DSSP as α-helices, β-sheets, turns, random coils, and unassigned regions. Topological domains derived from cryo-EM structural data are shown above each sequence segment. The red arrow indicates the position of the R230C substitution (residue 230). Overall, the WT and R230C systems exhibited highly similar secondary structure patterns throughout the simulation, indicating that the R230C variant does not induce major global alterations in the secondary structure organization of ABCA1.

The comparative analysis of secondary structure assignments along the full-length ABCA1 transporter revealed a high degree of structural conservation between the WT protein and the R230C variant throughout the MD simulation (Figure 3; Supplementary Figure S1). Consistent with this observation, the overall proportions of secondary structure elements remained highly similar between both proteins, with α-helices representing approximately 40.7% and 40.6%, β-sheets 6.4% and 6.2%, turns 14.4% and 13.9%, and coils 19.7% and 20.9% of the analyzed residues in the WT and R230C proteins, respectively (Supplementary Table S1). Despite this overall conservation, localized differences in the distribution of α-helices, β-sheets, turns, and random coils were observed. The most pronounced changes occurred within the N-terminal ECD1, surrounding residue 230. Specifically, the R230C variant exhibited an altered distribution of secondary structure elements between residues 220 and 250, characterized by a redistribution of coil, turn, and β-sheet conformations relative to the WT protein, while the overall helical architecture remained largely preserved. These changes suggest an increased tendency for local conformational rearrangements in the region containing the polymorphic site.

Additional differences in secondary structure occupancy were detected throughout ECD1 and adjacent regions of TMD1, where several residues displayed transitions between random coil and ordered conformations, including β-sheet and turn motifs. Although these variations were discontinuous and spatially restricted, they occurred more frequently in the R230C variant than in the ABCA1 WT, indicating that the substitution of arginine for cysteine influenced local structural flexibility. Similar localized alterations were also observed within the central region containing NBD1 and the TMD1–NBD1 interface, where subtle changes in the distribution of secondary structure elements were observed. Importantly, the structural perturbations associated with the R230C substitution remained localized and did not propagate into large-scale secondary structure rearrangements across the transmembrane domains or nucleotide-binding domains. Nevertheless, the increased frequency of local secondary structure transitions near residue 230 suggests that the polymorphism may modulate regional conformational dynamics, potentially affecting long-range intramolecular communication and the functional behavior of the transporter.

**Figure 4:**
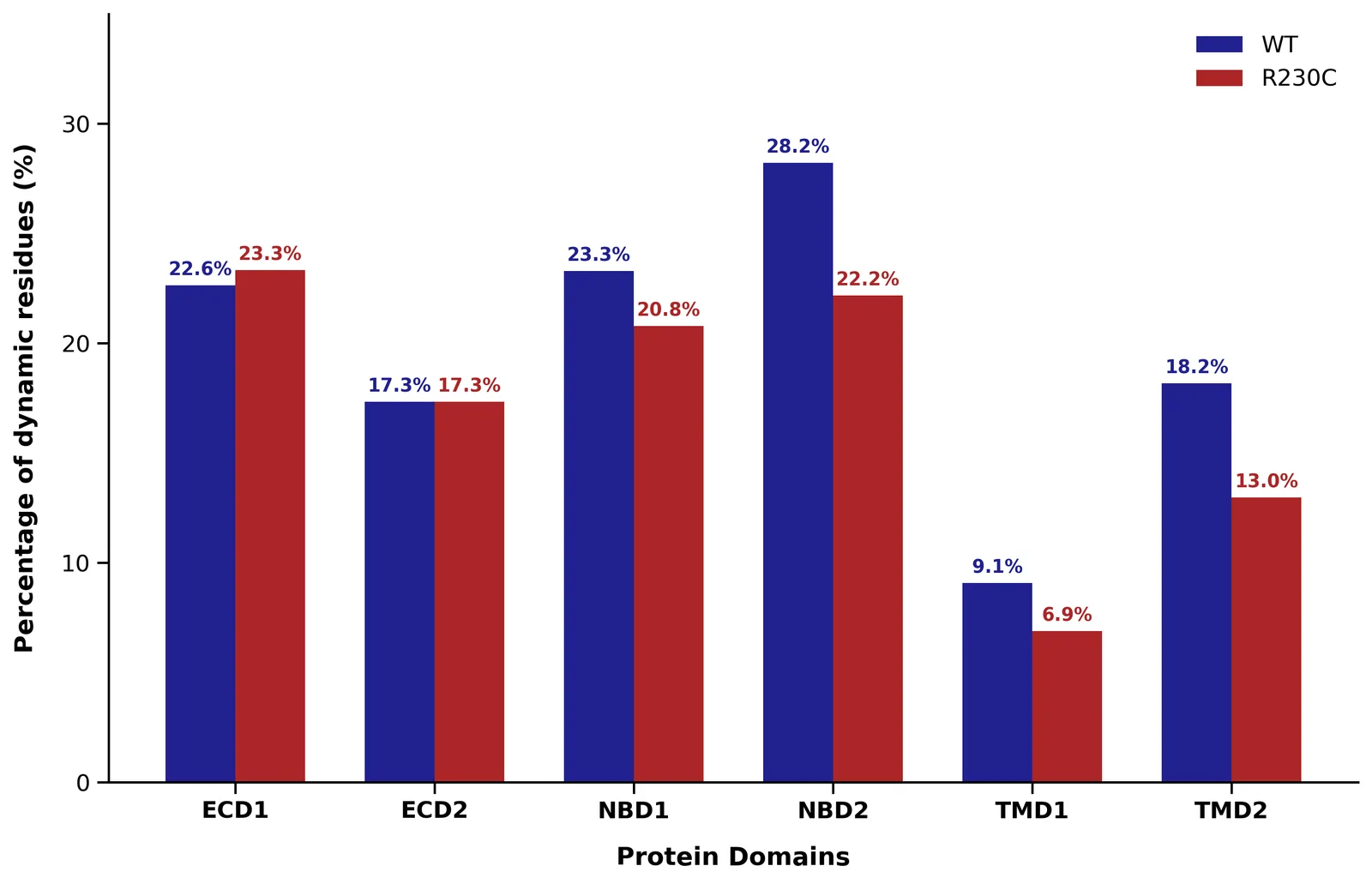
Domain-wise distribution of dynamic residues in ABCA1. The percentage of dynamic residues within each domain was calculated and compared between the wild-type protein and the R230C variant.

To further evaluate the impact of the R230C substitution on local conformational behavior, the proportion of residues classified as mixed/unassigned during the MD simulation was quantified for each major ABCA1 domain (Figure 4; Supplementary Table S2; Supplementary Figures S2 and S3). The analysis revealed domain-specific differences in the distribution of dynamic residues between the ABCA1 WT and R230C variants. The most pronounced reductions in dynamic residue content were observed in the nucleotide-binding domains and transmembrane regions. NBD2 exhibited the largest decrease, with the percentage of dynamic residues decreasing from approximately 28% in the WT protein to 22% in the R230C variant. Similarly, TMD2 showed a marked reduction from approximately 18% to 13%, while NBD1 and TMD1 decreased from 23% to 20% and from 9% to 7%, respectively. These findings suggest that the substitution of arginine for cysteine is associated with a lower degree of conformational heterogeneity in regions directly involved in ATP binding and transmembrane transport.

In contrast, ECD1 displayed a slight increase in the proportion of dynamic residues in the R230C variant relative to the ABCA1 WT, whereas ECD2 remained essentially unchanged. Notably, the slightly higher proportion of dynamic residues within ECD1 corresponds to the domain containing residue 230, supporting the secondary structure mapping analysis, which identified localized alterations in structural-state occupancy around the amino acid substitution site. Together, these results indicate that the R230C substitution promotes localized conformational flexibility near the N-terminal extracellular region while simultaneously reducing structural heterogeneity in distal functional domains, particularly NBD2 and TMD2.

### 2.2. Effects of the R230C Variant on Membrane Structural and Lipid Orientation Analysis

**Figure 5:**
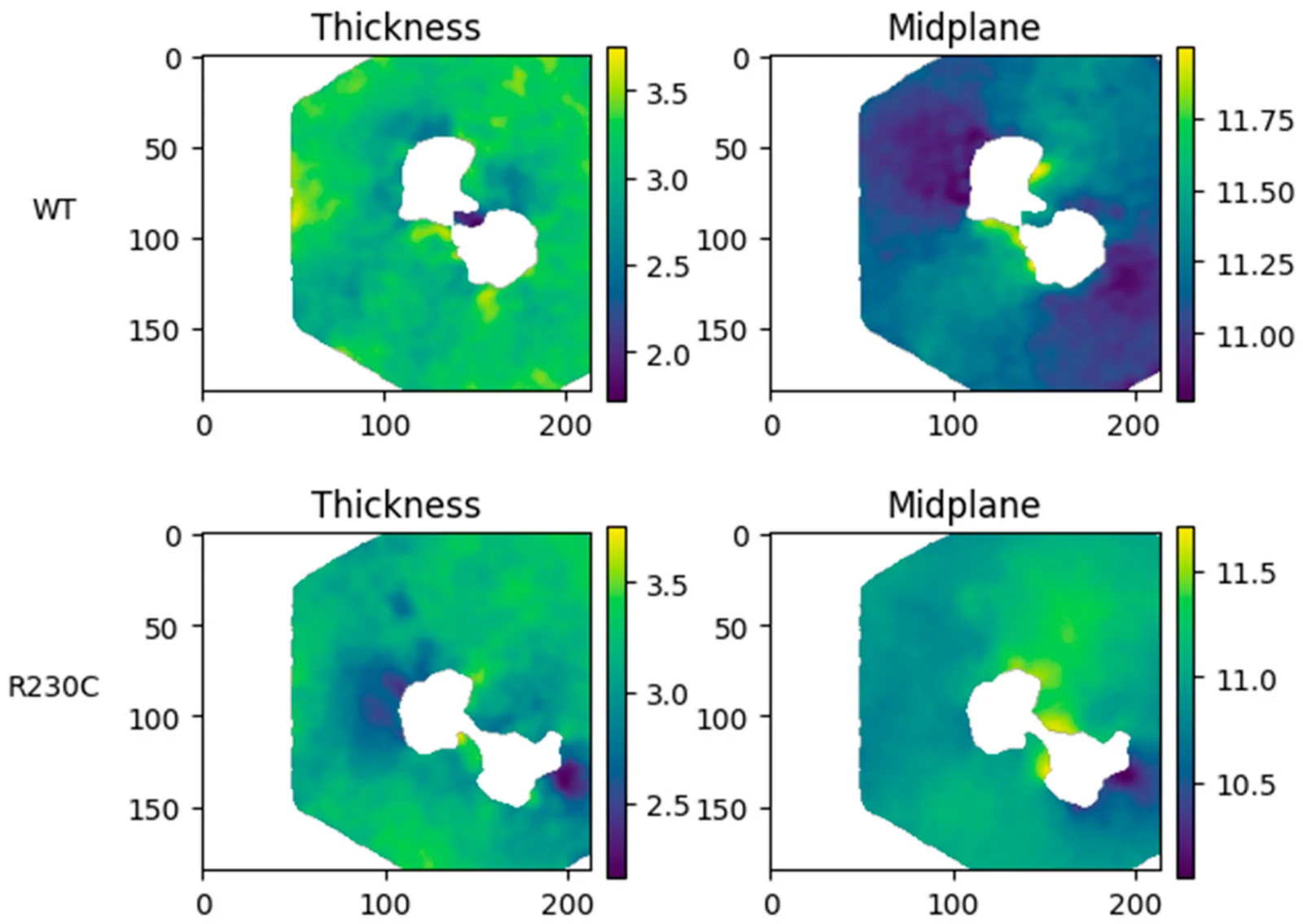
Membrane thickness and midplane maps surrounding ABCA1 during MDS. Two-dimensional maps of local membrane thickness (left panels) and membrane midplane position (right panels), given in nanometers (nm), averaged over the molecular dynamics trajectories for WT ABCA1 (upper panels) and the R230C variant (lower panels). The white regions correspond to the protein footprint within the lipid bilayer. Color gradients indicate spatial variations in membrane thickness and midplane position (scale values in nm), revealing local membrane deformations induced by the embedded transporter.

Membrane thickness and midplane maps were generated to characterize the lipid bilayer surrounding ABCA1 during the simulations (Figure 5). Both WT and R230C proteins exhibited localized variations in membrane thickness and midplane position around the transmembrane domains, whereas regions distant from the protein remained comparatively homogeneous. Compared with WT, the R230C variant exhibited a broader region of membrane thinning and a greater displacement of the membrane midplane around the transmembrane region. Furthermore, the spatial pattern of the midplane deformation differed from that observed in the WT protein, suggesting that the Arg230Cys substitution induces subtle remodeling of the local membrane architecture.

**Figure 6:**
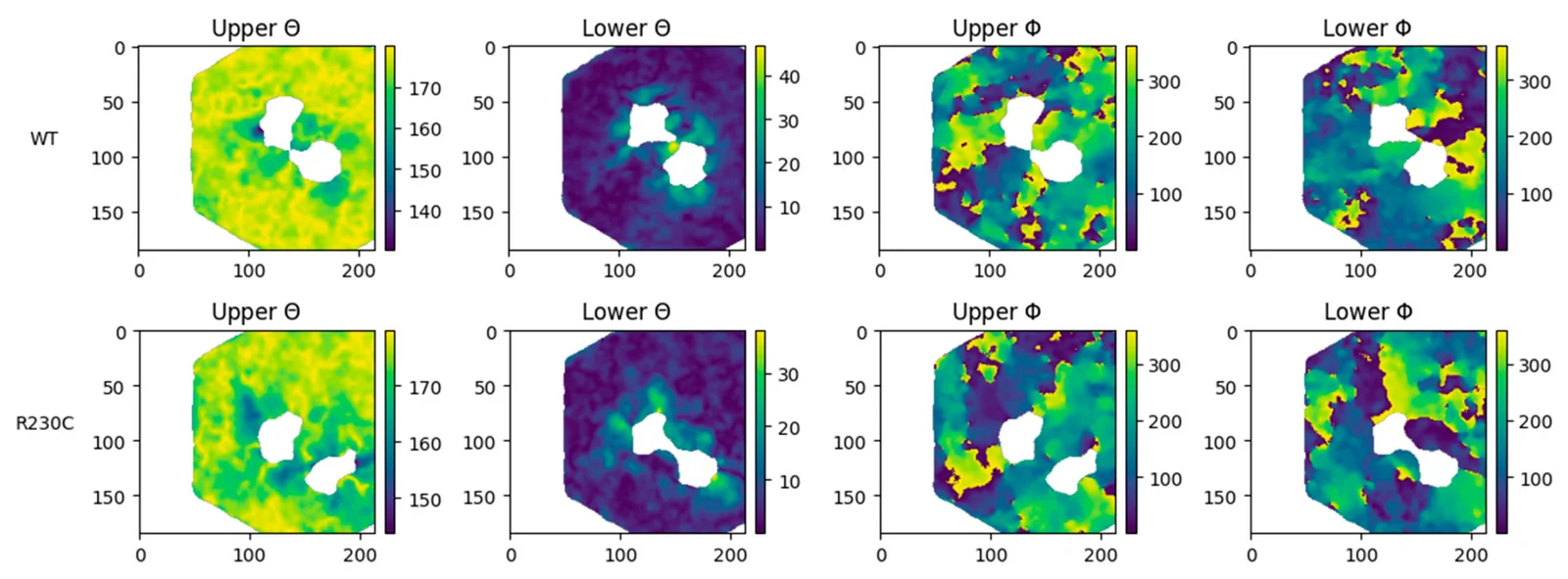
Lipid orientation maps of the upper and lower membrane leaflets surrounding wild-type (WT, top row) and R230C ABCA1 (bottom row) obtained from MD simulations. The Θ (theta) and Φ (phi) orientation angles, measured in degrees (°), are shown for both membrane leaflets. White regions correspond to the protein footprint projected onto the membrane plane, while the color scale represents the magnitude of each orientation parameter (in degrees), highlighting the spatial distribution of lipid orientation in the vicinity of the protein.

To evaluate the effect of the R230C variant on the organization of the surrounding lipid bilayer, lipid orientation maps (Θ and Φ) were generated for the upper and lower membrane leaflets (Figure 6; Supplementary Figure S4). Overall, both the WT and R230C proteins exhibited heterogeneous lipid orientation patterns, with localized perturbations surrounding the transmembrane region of ABCA1, whereas regions farther from the protein remained comparatively uniform. The Θ maps revealed relatively homogeneous lipid orientation in the upper leaflet for both proteins. However, the R230C variant showed a redistribution of localized low-Θ regions adjacent to the protein surface, suggesting subtle changes in the orientation of neighboring lipid molecules. In the lower leaflet, regions with elevated Θ values remained associated with the transmembrane domains in both proteins, although their distribution was altered in the R230C variant, indicating modified protein–lipid interactions. More pronounced differences were observed in the Φ maps. Compared with WT, the R230C variant exhibited a redistribution of lipid orientation domains in both membrane leaflets, particularly in the lower leaflet, where broader and more continuous Φ regions surrounded the transmembrane region, whereas the WT displayed a more fragmented pattern. These findings indicate that the Arg230Cys substitution modifies the local lipid orientational landscape without substantially altering the overall membrane organization rather than inducing large-scale membrane changes.

### 2.3. Dihedral Principal Component Analysis

**Figure 7:**
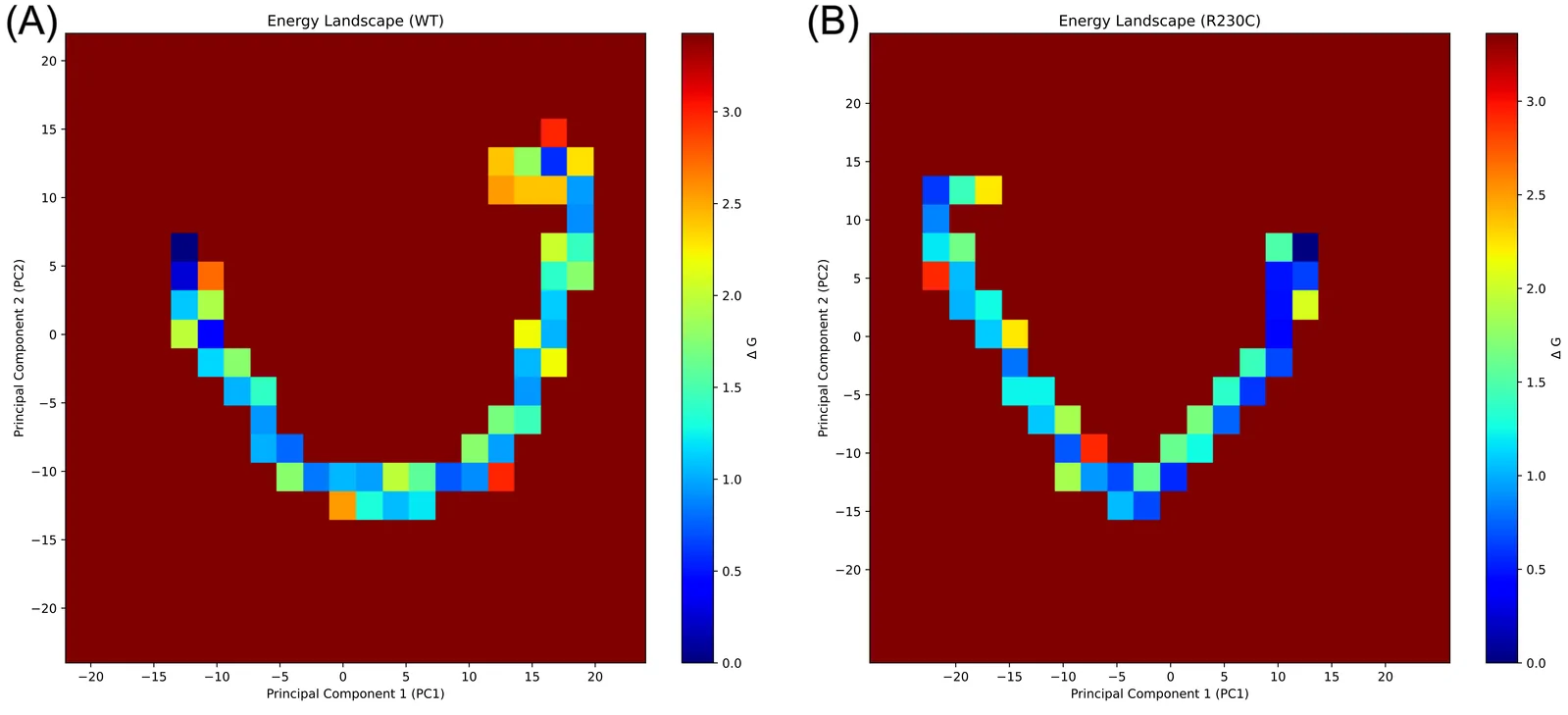
Free energy landscapes (FELs) of the human ABCA1 transporter projected onto the first two principal components (PC1 and PC2) derived from dihedral principal component analysis (dPCA). (**A**) ABCA1 WT and (**B**) R230C variant. The color-coded scale bar on the right represents the relative free energy (**Δ***G*), where dark blue zones indicate the global thermodynamic minima (**Δ***G* = 0.0), and the dark red matrix represents high-energy configurations (**Δ***G* > 3.5).

To isolate the high-amplitude collective internal motions and characterize the equilibrium thermodynamics of both the wild-type (WT) and R230C human ABCA1 transporters, FELs were constructed by projecting their trajectories onto the first two dihedral principal components (PC1 and PC2) (Figure 7). The ABCA1 WT exhibits a continuous, crescent-shaped free energy valley that spans an essential subspace from −13 to +20 along PC1, and from −13 to +15 along PC2 (Figure 7A). The thermodynamic global minima (**Δ***G* = 0.0, dark blue regions) for ABCA1 WT are localized at two opposite distal clusters: a highly negative PC1 pocket (PC1 ≈ −12, PC2 ≈ 5) and a highly positive PC cluster (PC1 ≈ 17, PC2 ≈ 12). These two deep basins are connected via a continuous, uninterrupted low-energy corridor characterized by accessible intermediate states with free energy values ranging from 1.0 to 2.0.

In contrast, the introduction of the R230C mutation induces a distinct shift and narrowing in the sampled conformational phase space (Figure 7B). The R230C variant samples a V-shaped landscape that is markedly shifted toward negative coordinates along the first principal axis, ranging from −23 to +14 on PC1, and from −15 to +14 on PC2. Furthermore, the distribution of the global minima (**Δ***G* = 0.0) in the R230C variant undergoes structural re-partitioning; the deepest basins are fragmented into three segregated pockets located at the upper-left terminal (PC1 ≈ −22, PC2 ≈ 13), the lowermost turn (PC1 ≈ −3, PC2 ≈ −14), and the upper-right branch ≈ 8) of the V-shaped landscape. The intermediate regions bridging these basins display more defined boundaries and a localized high-energy barrier (**Δ***G* ≈ 3.0, red square) situated along the left arm (PC1 ≈ −7, PC2 ≈ −9) that is absent in the WT landscape.

To explicitly map internal dynamic coupling across the transporter, 2D backbone dihedral variance-covariance heatmaps were generated from the dPCA trajectories (Supplementary Figure S5). In WT ABCA1, the covariance matrix displays extensive, continuous off-diagonal correlation blocks connecting the extracellular domain 1 (ECD1) with distal transmembrane and nucleotide-binding domains (Supplementary Figure S5A). In contrast, the R230C variant exhibits a pronounced re-partitioning and attenuation of off-diagonal covariance features (Supplementary Figure S5B). This matrix-level shift demonstrates that the R230C substitution alters long-range internal communication, providing direct structural evidence for allosteric decoupling between the N-terminal loop and distal catalytic centers.

### 2.4. Tunnel Analysis

**Figure 8:**
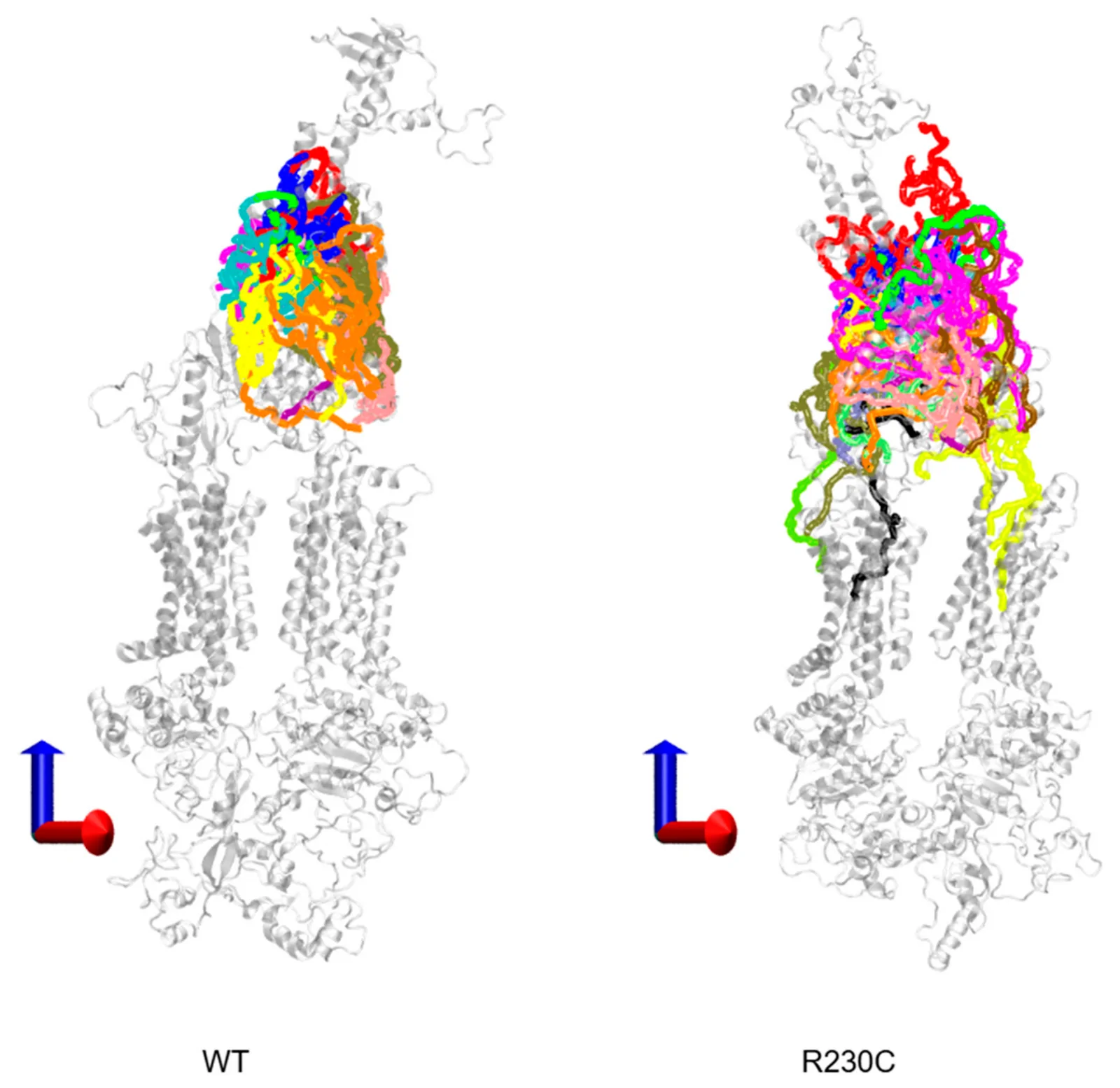
Representative CAVER tunnel analysis of ABCA1 WT and the R230C variant. The protein backbone is shown as a transparent gray cartoon, and the colored traces represent individual tunnel clusters identified from the molecular dynamics trajectories. The WT protein (left) contained 10 tunnel clusters, whereas the R230C variant (right) exhibited 17 tunnel clusters, revealing differences in the spatial distribution and organization of the predicted internal transport pathways. Coordinate axes indicate spatial orientation: the vertical blue arrow represents the z-axis (membrane normal), the red arrow represents the x-axis, and the green arrow (y-axis) is aligned along the line of sight behind the x-axis.

To investigate whether the arginine substitution for cysteine at the 230 residue affects the putative intramolecular transport pathways of ABCA1, tunnel analysis was performed using CAVER on the molecular dynamics trajectories. In both the ABCA1 WT and R230C variant, multiple tunnels were identified within the extracellular domain (Figure 8), forming interconnected pathways that extend toward the transmembrane region and are consistent with the hydrophobic cavity proposed to participate in cholesterol transport.

CAVER analysis revealed marked structural and dynamic differences in the internal transport pathways between WT ABCA1 and the R230C variant (Figure 8; Supplementary Tables S3 and S4). While WT ABCA1 contained 10 tunnel clusters, the R230C variant displayed 17 distinct clusters. Crucially, quantitative evaluation of individual cluster metrics demonstrates that the primary transport conduits are directly compromised in the R230C variant. In WT ABCA1, the two dominant functional pathways (Clusters 1 and 2 in Supplementary Table S3) maintained 100% frame persistence across all analyzed trajectory snapshots (20/20 frames), exhibiting high throughput priority indices (0.692 and 0.668), wide bottleneck radii (1.695 Å and 1.488 Å), and short path lengths (19.82 Å and 14.64 Å). In contrast, in the R230C variant (Supplementary Table S4), these primary functional conduits showed reduced frame persistence (85% for Cluster 1; 75% for Cluster 2), indicating intermittent channel closures. Correspondingly, throughput priority dropped to 0.556 for Cluster 1 and 0.490 for Cluster 2 (representing a 26.6% reduction for Cluster 2 and a 23.1% combined throughput reduction across the primary path system). Additionally, the primary bottleneck in Cluster 1 narrowed to 1.587 Å, while path lengths extended to 22.32 Å (Cluster 1) and 23.07 Å (Cluster 2).

Furthermore, the increase in overall tunnel clusters in R230C (from 10 to 17) was driven by the appearance of low-persistence, transient secondary pathways. While WT ABCA1 contained only a single minor cluster detected in fewer than 10 snapshots (Supplementary Table S3), the R230C variant exhibited 7 transient clusters detected in 10 or fewer snapshots (Supplementary Table S4). These secondary pathways displayed markedly elongated path lengths, reaching up to 183.31 Å (Cluster 17, detected in only a single snapshot). Rather than providing redundant functional routes, these transient channels reflect pathway fragmentation. Complete geometric and dynamic parameters for all identified tunnel clusters in WT and R230C are summarized in Supplementary Tables S3 and S4, respectively.

## 3. Discussion

The present MDS study demonstrates that substitution of arginine by cysteine at position 230 does not produce large-scale structural disruption or destabilization of ABCA1 protein but instead induces subtle alterations in its conformational dynamics. The replacement of a large, positively charged arginine residue with a smaller, uncharged cysteine represents a substantial physicochemical change that is expected to modify the local interaction network surrounding residue 230. Arginine is capable of establishing multiple electrostatic interactions and hydrogen bonds that contribute to structural stabilization, whereas cysteine possesses a polar thiol group with reduced hydrogen-bonding capacity and distinct chemical properties. Consistent with these differences, collectively the molecular dynamics analyses suggest that the R230C substitution reshapes the dynamic landscape of ABCA1 by modifying local flexibility around the 230 residue while simultaneously altering the conformational behavior of distal functional domains without compromising the overall structural architecture of the membrane cholesterol transporter.

In this work, our computational model was based on the high-resolution cryo-EM structure of human ABCA1 in its basal, nucleotide-free state (PDB ID: 5XJY) [10,15]. While this state captures the baseline physiological conformation embedded in the lipid bilayer and reveals how the R230C variant natively alters ECD1 dynamics, tunnel organization, and allosteric coupling to distal NBD/TMD domains prior to power-stroke activation, ABCA1 functionally transitions through multiple conformational states during its ATP-dependent transport cycle. Exploring ATP-bound or NBD-dimerized conformations in future simulations could provide complementary insights into how R230C influences downstream power-stroke transitions and active cholesterol translocation.

From a methodological standpoint, simulating full-length ABCA1 embedded in a multi-component lipid raft bilayer presents significant computational challenges due to the extensive conformational space inherent to large ABC transporters. In this study, to isolate the specific biophysical impact of the R230C variant, both WT and mutant systems were evaluated using a strictly standardized, comparative simulation setup originating from identical initial coordinates, membrane environments, and forcefield parameters. The multi-parameter convergence observed across independent descriptors—including free energy landscape partitioning, distal domain rigidification, and primary tunnel throughput reduction—indicates that the observed differences reflect a genuine, mutation-induced shift in protein dynamics rather than baseline stochastic drift. While this standardized approach successfully captures the baseline differential response in the nucleotide-free state, future simulations incorporating independent replicas and active, ATP-bound functional states will be valuable to further map the broader kinetic landscape and long-time conformational transitions of ABCA1 variants.

The RMSD analysis indicates that the R230C substitution modifies the conformational dynamics of ABCA1 without affecting its overall structural stability. Although both proteins reached similar equilibrium during the early stages of the simulation, the ABCA1 WT protein exhibited greater conformational diversity during the final 80 ns, whereas the R230C variant remained confined to a narrower range of conformations, defined for the lower RMSD values. These findings indicate that replacement by Cys decreased global structural stability and potentially limited the conformational structural transitions required for the cholesterol efflux at the plasma membrane. In addition, the RMSF analysis further supports this interpretation. Although the overall flexibility profiles remained highly similar between both systems, several regions exhibited reduced residue mobility in the R230C variant. The preservation of the principal flexibility hotspots indicates that the mutation does not globally perturb the intrinsic dynamics of ABCA1. Instead, the localized reduction in fluctuations across multiple regions suggests that the substitution introduces selective stabilization of specific structural segments. Such localized reduction in conformational flexibility (rigidification) is consistent with the lower RMSD observed during the later stages of the trajectory and suggests that the mutation constrains local motions rather than altering the overall fold of the transporter.

Interestingly, this apparent increase in local rigidity occurs simultaneously with an increase in the radius of gyration and solvent-accessible surface area. At first glance, these observations may appear contradictory, since lower flexibility is often associated with greater structural compactness. However, these parameters describe different aspects of protein behavior. Whereas RMSD and RMSF primarily reflect conformational fluctuations, Rg and SASA describe the global spatial organization of the protein. Accordingly, the R230C variant appears to adopt a slightly more expanded conformation while exhibiting reduced internal conformational mobility. This combination suggests that the substitution stabilizes a more open structural state rather than increasing structural disorder. The convergence of SASA values after approximately 120 ns further indicates that this expansion for solvent exposition primarily affects the early stages of conformational adaptation while the protein is still relaxing from its initial structure toward a more energetically favorable conformation. Subsequently, both proteins reach a relatively stable equilibrium, in which their structural properties undergo only minor fluctuations over time.

The functional relevance of preserving the structural integrity of the extracellular domains is supported by recent experimental evidence showing that missense variants affecting these regions significantly impair ABCA1-mediated cholesterol efflux, underscoring the critical role of extracellular domain architecture in transporter activity [23]. These findings are consistent with a previous molecular dynamics study showing that ABCA1 is an intrinsically dynamic transporter whose extracellular domains undergo substantial conformational fluctuations that are essential for lipid transport [15]. Since residue 230 is located within ECD1, substitution of the positively charged arginine by the smaller, neutral cysteine is likely to alter the local interaction network and modulate long-range allosteric communication throughout the protein. Consequently, the reduced conformational sampling observed for the R230C variant may impair the structural plasticity required for the coordinated ATP-dependent conformational transitions that drive free cholesterol and phospholipid efflux to apoA-I [21], thereby reducing the efficiency of nascent HDL biogenesis and providing a plausible molecular explanation for the decreased transporter activity associated with this variant.

Secondary structure analysis further elucidated the structural effects of the amino acid substitution. Although the overall secondary structure of ABCA1 remained highly conserved throughout the simulations, localized changes were consistently observed around residues 220–250, precisely where Arg230 is replaced by cysteine. In the R230C variant, this region exhibited a redistribution of coil, turn, and β-sheet elements, suggesting that the loss of the positively charged arginine modifies the local structural equilibrium. Because arginine frequently contributes to stabilizing secondary structure through electrostatic interactions and hydrogen-bond networks, its substitution by cysteine likely alters local intramolecular contacts, thereby increasing the frequency of conformational transitions within ECD1. In addition, the introduction of a cysteine residue introduces the possibility of thiol-mediated interactions, including disulfide bond formation under oxidative conditions. Given that the extracellular domains of ABCA1 contain several conserved disulfide bonds that are essential for structural integrity, the presence of Cys230 may further influence the local conformational environment, and could warrant further investigation of its local extracellular environment. Although no disulfide bond formation was observed or modeled during the present simulations. Importantly, these alterations remained spatially restricted and did not propagate into major rearrangements of the transmembrane helices or nucleotide-binding domains.

Although no major secondary-structure rearrangements were detected outside ECD1, analysis of dynamic residue distributions revealed effects extending to distal functional domains. Quantification of dynamic residues demonstrated a marked reduction in conformational heterogeneity within both nucleotide-binding domains and transmembrane domains, particularly NBD2 and TMD2. These findings suggest that replacement of Arg230 by Cys230 modifies long-range allosteric communication throughout the transporter. ABC transporters function through highly coordinated interactions between extracellular domains, transmembrane helices, and ATP-binding cassettes; therefore, even subtle perturbations introduced within ECD1 may propagate through the protein and alter the dynamic coupling required for ATP-dependent conformational cycling.

These observations are consistent with the known biological effects of the R230C polymorphism. Experimental studies have shown that carriers of the C230 alleles exhibit reduced plasma HDL cholesterol levels [17,18,19] and impaired ABCA1-mediated cholesterol efflux despite relatively preserved protein expression [16]. Our simulations provide a structural explanation for these findings by showing that the R230C substitution does not destabilize ABCA1 globally but instead shifts its conformational equilibrium through local structural perturbations. Increased flexibility around Cys230 in ECD1 was accompanied by reduced conformational heterogeneity in the nucleotide-binding and transmembrane domains, suggesting altered long-range allosteric communication. This redistribution of conformational dynamics could impair the coordinated ATP-driven conformational transitions required for efficient lipid transport, thereby reducing ABCA1 activity.

The plasma membrane is not merely a passive environment for ABC transporters but actively contributes to their structural dynamics and transport cycle. Increasing evidence indicates that protein–lipid interactions and the physical properties of the surrounding bilayer, including membrane thickness, curvature, and lipid organization, modulate the conformational transitions required for substrate translocation [24]. Consistent with this concept, our simulations revealed that although both ABCA1 WT and the R230C variant induced localized perturbations in the surrounding lipid bilayer, the R230C substitution redistributed these perturbations without inducing global membrane remodeling.

The membrane thickness and midplane analyses demonstrated that the effects of both proteins were largely confined to the vicinity of the transmembrane domains, consistent with previous molecular dynamics studies showing that membrane deformations induced by integral membrane proteins are highly localized [15,21]. However, the broader membrane thinning and altered midplane displacement observed around the R230C variant suggest that the arginine substitution for cysteine induced subtly changes the mechanical coupling between ABCA1 and the surrounding bilayer. Such local membrane remodeling may reflect changes in the interactions between the transmembrane helices and surrounding lipids, thereby influencing the conformational dynamics of ABCA1. The lipid orientation maps further support this interpretation. Although the global organization of the membrane remained largely preserved, the redistribution of Θ and, particularly, Φ orientation domains indicates that the R230C substitution alters the local orientational environment of membrane lipids; these effects were most evident in the lower leaflet, where broader and more continuous orientation domains surrounded the 230 residue. Rather than indicating increased membrane disorder, these observations suggest a reorganization of lipid packing and orientation at the protein–lipid interface, consistent with modified interactions between ABCA1 and its surrounding lipid molecules, that may contribute to the different conformational dynamics of the transporter observed in the R230C variant.

The observed membrane remodeling is mechanistically plausible considering the physicochemical properties of the substituted residue. Arginine is a positively charged amino acid capable of establishing electrostatic interactions and hydrogen bonds with phospholipid headgroups, whereas cysteine is smaller, neutral under physiological conditions, and considerably less effective at stabilizing such interactions. Although residue 230 is located outside the transmembrane region, previous structural and computational studies have demonstrated that mutations distant from membrane-embedded helices can propagate their effects through long-range allosteric communication, ultimately altering transmembrane dynamics and protein–lipid interactions [21,25]. Therefore, the changes observed in the local membrane environment likely arise from polymorphism-induced alterations in the conformational behavior of ABCA1 rather than from direct disruption of lipid contacts at residue 230.

The modified lipid environment observed here may therefore represent both a consequence and a contributor to these altered protein dynamics. Because membrane deformation and lipid rearrangement are closely coupled to the functional motions of ABC transporters, subtle changes in the local bilayer organization could influence the conformational transitions required for phospholipid and cholesterol translocation. Although the lipid membrane analyses are qualitative, they consistently indicate that the R230C variant remodels the membrane microenvironment surrounding ABCA1 while preserving the overall architecture of the lipid bilayer. This behavior is in agreement with the functional phenotype previously reported for the R230C polymorphism, which impairs cholesterol efflux [16] and is associated with reduced plasma HDL-Cholesterol levels [17,18], despite normal membrane localization of the transporter. The present simulations therefore suggest that the functional consequences of R230C may arise not from major structural destabilization but from subtle modifications in the dynamic coupling between ABCA1 and its surrounding membrane, which could alter the efficiency of the cholesterol transport cycle.

These observations are further supported by complementary membrane analyses (Supplementary Figures S6–S8). Lipid tail order parameter maps showed slightly broader regions of reduced lipid order surrounding the R230C protein, consistent with localized perturbations in lipid packing. In addition, cholesterol-contact analysis revealed that the R230C substitution redistributed cholesterol interaction sites along the transporter while reducing the maximum interaction frequency at individual residues. Interestingly, the interaction sites are focalized in TM1, TM10, TM11 and EH1 for WT, all of them close together forming a gate of sorts, whereas for R230C some interactions are observed away from this region in the outer surface of the TM domain (TM3); some regions that show a large number of interactions for WT show no interactions at all for R230C (F1758, V1771) and some interactions are observed well beyond the aforementioned “gate”, deep within the inner space of the TM domain (Y648 in TM2, F753 in TM5). Together, these complementary findings reinforce the notion that the Arg230Cys substitution primarily remodels the local protein–lipid environment rather than inducing large-scale changes in membrane architecture. Further quantitative analyses integrating membrane order parameters, lipid residence times, dynamic cross-correlation, residue interaction networks together with experimental validation, will be valuable to establish how these local membrane alterations influence the molecular mechanism of cholesterol export mediated by ABCA1.

The molecular mechanism of ABCA1-mediated lipid translocation relies on tightly coordinated allosteric transitions that couple ATP binding and hydrolysis to large-scale conformational changes required for cholesterol transport [26]. Using dihedral principal component analysis (dPCA) [27], we mapped the free energy landscapes (FELs) of ABCA1 WT and the R230C variant (Figure 7). The continuous, crescent-shaped free energy corridor observed for ABCA1 WT reveals a highly cooperative, low-barrier thermodynamic pathway. Two major global minima connected by an uninterrupted valley (**Δ***G* ≈ 1.0–2.0 kcal/mol) indicate that the native transporter retains the intrinsic flexibility required to transition efficiently between functional conformational states during lipid translocation. In contrast, the R230C substitution reshapes this cooperative landscape into a narrower, V-shaped topology and partitions the free energy landscape into three isolated low-energy conformational basins. These basins represent distinct stable conformational ensembles separated by higher-energy barriers, suggesting impaired long-range allosteric communication and restricting the transporter to more segregated conformational states. A key feature of the mutant landscape is the emergence of a localized high-energy barrier (**Δ***G* ≈ 3.0 kcal/mol) along the left branch (PC1 ≈ −7, PC2 ≈ −9), a kinetic obstacle absent in WT. At the molecular level, replacement of the bulky, positively charged arginine with a smaller, neutral cysteine likely disrupts local electrostatic interactions or salt bridges, limiting conformational transitions. These findings provide a mechanistic explanation for the loss-of-function phenotype associated with the R230C variant and its association with hypoalphalipoproteinemia and metabolic diseases [18,20]. By becoming kinetically trapped, the mutant transporter is less able to complete the conformational cycles required for cholesterol and phospholipid translocation, thereby impairing nascent HDL biogenesis.

Mechanistically, the long-range allosteric crosstalk between the ECD1 polymorphic site and distal domains is directly illustrated by the 2D backbone dihedral covariance matrices (Supplementary Figure S5). By evaluating internal dihedral space (*ϕ*, *ψ*) rather than Cartesian coordinates, this analysis isolates pure internal structural coupling free from global superposition artifacts. The transition from broad, interconnected covariance blocks in WT to a fragmented, attenuated matrix in R230C (Supplementary Figure S5B) explains how localized fluctuations near Cys230 induce downstream dynamic rigidification in NBD2 (21.4% reduction) and TMD2 (27.8% reduction; Supplementary Table S2), ultimately altering the global free energy landscape (Figure 7).

Rather than merely altering overall tunnel count, the R230C substitution specifically degrades the geometric integrity, continuity, and throughput capacity of the primary functional cholesterol transport pathway. In WT ABCA1, substrate translocation is facilitated by a highly persistent, high-throughput, and spatially direct hydrophobic corridor (Supplementary Table S3). In the R230C variant, this primary conduit undergoes bottleneck narrowing, pathway extension, and intermittent closure across simulation frames (Supplementary Table S4). Concurrently, local conformational fluctuations in ECD1 promote the transient opening of multiple long, tortuous secondary tunnels.

Quantitatively, the combined throughput priority of the primary transport conduits drops by 23.1% (with Cluster 2 showing an individual reduction of 26.6%) in R230C compared to WT (Supplementary Tables S3 and S4). Mechanistically, these transient secondary channels act as non-productive “decoy” routes that induce spatial dispersion and elevate kinetic barriers for translocating sterols. A cholesterol molecule entering the extracellular cavity of R230C is therefore less likely to traverse a continuous, high-throughput functional conduit and more likely to become trapped in transient blind alleys. This 23.1–26.6% reduction in calculated geometric throughput capacity provides a direct biophysical basis that closely accounts for the 27% reduction in cholesterol efflux observed experimentally in clinical and functional studies of the R230C variant [16]. These observations align with recent computational studies demonstrating that missense variants in ABCA1 can induce localized structural perturbations capable of altering interactions with ApoA-I and compromising cholesterol transport efficiency [28].

Cryo-electron microscopy studies identified a hydrophobic tunnel extending through the extracellular domains of human ABCA1, providing structural evidence for a putative pathway which has been proposed to mediate the translocation of cholesterol and phospholipids toward apolipoprotein A-I during nascent HDL formation [10]. In the WT protein, the detected tunnel clusters remained closely associated with this hydrophobic cavity, consistent with the presence of a relatively well-defined preferential transport pathway. In contrast, the R230C variant redistributed the tunnel network into multiple alternative routes extending away from the canonical hydrophobic tunnel. Although CAVER identifies geometrically accessible pathways rather than substrate movement, this reorganization suggests that the mutation may reduce the persistence of a preferential transport route, thereby decreasing the efficiency of directional cholesterol translocation. This interpretation is consistent with experimental studies showing that the R230C polymorphism reduces ABCA1-mediated cholesterol efflux [16] despite preserving membrane localization. Together with the observed changes in conformational dynamics and protein–lipid interactions, the altered tunnel organization provides a plausible structural mechanism linking the Arg230Cys substitution to the reduced cholesterol transport efficiency associated with this variant.

Overall, the present results support the concept that the R230C variant acts primarily as a dynamic allosteric modulator rather than a structurally disruptive mutation. The physicochemical differences between arginine and cysteine reshape the local conformational environment surrounding residue 230, while preserving the global architecture of ABCA1. These localized perturbations propagate through long-range allosteric communication, ultimately reducing conformational heterogeneity within domains that drive cholesterol transport. Together, these findings provide a plausible molecular mechanism linking the R230C variant with the functional impairment previously described for ABCA1 and highlight the importance of protein dynamics in understanding the pathogenic effects of naturally occurring missense variants. Further computational studies integrating principal component analysis, residue interaction network analysis and free-energy landscape reconstruction together with complementary experimental validation, will provide a more comprehensive understanding of how the R230C variant reshapes the conformational landscape of ABCA1 and influences the coordinated structural transitions underlying the cholesterol and phospholipids transport mediated by ABCA1 and its contribution to the pathophysiology of metabolic diseases.

## 4. Materials and Methods

### 4.1. Protein Sequence

The structural template used for computational modeling was the cryo-electron microscopy (cryo-EM) structure of the human ATP-binding cassette transporter A1 (ABCA1) (PDB ID: 5XJY). This structure was determined at an overall resolution of 4.1 Å, with the extracellular domain resolved at approximately 3.9 Å, and represents ABCA1 in a nucleotide-free outward-facing conformation, in which the transmembrane, nucleotide-binding, and extracellular domains form an extended hydrophobic tunnel. The amino acid sequence of the human wild-type ABCA1 protein (2261 amino acids) was retrieved from the UniProt database (accession O95477).

### 4.2. Molecular Dynamic Simulation

#### 4.2.1. Structural Modeling and Secondary Structure Analysis

Aiming for the evaluation of the effect of the R230C (rs9282541) SNP on the ABCA1 transporter, structural models of the wild-type and R230C mutant generated using CHARMM-GUI [29], were embedded in a lipid bilayer mimicking a lipid raft composition consisting of over 10 different lipids including POPC (64.3%), PSM (14.5%), cholesterol (6.7%), POPI (4.9%), POPE (3.9%), POPS (3.2%) and LLPC (2.5%). Optional modeling of 22 N-terminal residues and 50 C-terminal residues was omitted, so that both models range from Cys3 to Ser2234. MDS were carried out using GROMACS for 200 ns under identical conditions for both systems. The system was placed in a hexagonal periodic box with a height of 25.78854 nm and a base side length of 7.50753 nm, solvated with 123,350 TIP3P water molecules, and neutralized with 348 K^+^ and Cl^−^ ions. Following energy minimization, simulations were conducted for 10 ns under NPT condition at 303.15 K. Trajectory analysis was performed using GROMACS to assess structural stability and mutation-induced changes in secondary structure over time through comparative analysis of the wild-type and mutant systems. Secondary structure assignment was performed using the DSSP algorithm implemented in GROMACS. For comparative analysis and visualization, ABCA1 domain boundaries were defined according to Qian et al. (2017) [10]. DSSP secondary structure assignments were subsequently grouped into four major categories: helices (α-helix, 3_10_-helix, and π-helix), strands (extended β-strand and β-bridge), turns, and coils (coil and bend).

#### 4.2.2. Technical Description of the Data Visualization Process

To generate the high-resolution visual architecture, an automated plotting pipeline was developed in Python 3.0 using the object-oriented framework of Matplotlib 3.10. The computational workflow performed a deterministic linear partitioning (chunking) of the ABCA1 transporter primary sequence into contiguous 500-residue windows to minimize scale distortion and enhance graphical readability. To improve computational efficiency and reduce Python iteration overhead, biological data were vectorized into contiguous-memory NumPy arrays, and a hash table–based reindexing strategy was implemented to enable constant-time (O(1)) conformational lookups. Secondary structure profiles obtained from DSSP and Cryo-EM–derived topological domains were subsequently rendered as continuous, solid geometric patch maps devoid of separation artifacts (micro-gaps). In addition, vector-based allosteric markers were incorporated with pixel-level precision to accurately delineate the positional R230C ABCA1 variant while preventing label overlap and preserving annotation clarity.

#### 4.2.3. Membrane Structural and Lipid Orientation Analysis

To characterize the local perturbations and structural deformations induced by wild-type and R230C ABCA1 on the surrounding lipid bilayer, two-dimensional spatially resolved grid analysis was performed using the MOSAICS software suite [30]. Trajectory snapshots were mapped onto a two-dimensional spatial grid defined across the membrane plane (xy-plane). Local membrane thickness and midplane position maps were computed by measuring the average vertical positions and displacements of lipid headgroup phosphorus atoms in the upper and lower leaflets relative to the bilayer center. Grid cells corresponding to the spatial footprint occupied by the protein’s transmembrane domain were masked out to isolate the lipid environment.

Furthermore, local lipid orientational dynamics were evaluated independently for the upper and lower membrane leaflets. The orientation of surrounding lipid molecules was quantified using two angular parameters: the polar angle (Θ), defined as the tilt angle of the lipid acyl chains relative to the membrane normal, and the azimuthal angle (Φ), describing the in-plane orientational preference. The resulting spatially resolved property maps for membrane thickness, midplane deformation, and lipid tilt/orientation parameters (Θ and Φ) were averaged over the molecular dynamics trajectories and rendered as continuous two-dimensional color-coded profiles to evaluate localized membrane remodeling induced by the R230C variant.

#### 4.2.4. Principal Component Analysis

Dihedral principal component analysis (dPCA) was conducted to isolate and characterize the high-amplitude, internal collective motions of the human ABCA1 transporter from the molecular dynamics trajectories. Unlike standard Cartesian PCA, dPCA uses internal coordinates (dihedral angles), which naturally decouple internal structural fluctuations from overall rigid-body translation and rotation without requiring least-squares structural superposition. All dPCA calculations were executed using the CARMA software package [31].

To eliminate the periodicity artifacts inherent to angular variables—specifically phase jumps at boundary angles—each backbone dihedral angle (*ϕ* and *ψ*) across the analyzed residue sequence was mapped onto a continuous two-dimensional metric space using sine and cosine vector transformations. For a system containing N backbone dihedral angles, this transformation yields a 2N-dimensional coordinate space. A 2N × 2N positional covariance matrix was subsequently generated in CARMA by calculating the ensemble-averaged variances and covariances of these transformed metric coordinates across all analyzed trajectory snapshots.

Diagonalization of the covariance matrix yielded a set of orthogonal eigenvectors (principal components) describing the collective internal modes of structural motion, alongside their corresponding eigenvalues, which measure the variance captured along each principal axis. Eigenvectors were ordered by decreasing eigenvalue magnitude. Trajectory projections onto the low-dimensional subspace spanned by the first two principal components (PC1 vs. PC2) were used to construct free energy landscapes, allowing for the direct comparison of the essential dynamic phase space sampled by the wild-type and R230C systems and the assessment of allosteric dynamics induced by the functional variant. Finally, representative average structures for both wild-type and R230C ABCA1 were calculated and extracted from the PC1 vs. PC2 projections to conduct comparative structural analyses of their predominant conformational states.

#### 4.2.5. Analysis of Tunnel Dynamics

The transport of cholesterol involves, among other regions of ABCA1, a hydrophobic tunnel. As is commonplace in most proteins, ABCA1 shows several cavities and tunnels. Internal transport pathways and cavity dynamics within the extracellular and transmembrane domains of wild-type and R230C ABCA1 were characterized using the CAVER 3.0 software package [32]. Calculations were performed on 20 trajectory snapshots extracted at regular time intervals across the 200 ns molecular dynamics simulations.

The starting origin for tunnel detection was defined at the center of the conserved hydrophobic extracellular cavity situated between ECD1 and ECD2, corresponding to the putative pathway implicated in cholesterol transport toward ApoA-I. Tunnel computation was conducted using a minimum probe radius of 0.5 Å, a shell radius of 4.5 Å, and a shell depth of 4.0 Å. To account for conformational fluctuations during the trajectory, detected pathways across all analyzed snapshots were grouped into spatial tunnel clusters using hierarchical average-linkage with a clustering threshold of 20.

For each identified tunnel cluster, geometric and dynamic transport descriptors were calculated and evaluated, including:

- Bottleneck radius (Å): Defined as the maximum radius of the narrowest bottleneck along the internal pathway.
- Tunnel length (Å): Measured as the spatial distance from the internal starting origin to the protein surface.
- Throughput: An integrated metric combining bottleneck radius and pathway length to assess overall transport probability.
- Persistence: Evaluated as the frequency of occurrence of a given tunnel cluster across the analyzed snapshot ensemble.

Spatial distributions and representations of the resulting tunnel networks were rendered using CAVER visualization modules to compare the persistence, structural organization, and pathway heterogeneity between WT and R230C ABCA1.

The pathway cholesterol molecules are supposed to follow from the membrane to the interaction site with ApoA-I is not wide open in the ATP-free structure. Nevertheless, a few tunnels were found in the WT MDS that are in agreement with the current model of cholesterol transport. No such tunnels were found in the R230C MDS, while other tunnels appeared, which suggests a perturbation in the tunnels required for cholesterol transport is introduced by the mutation.

## 5. Conclusions

The present study demonstrates that the ABCA1 R230C variant does not disrupt the overall architecture of the transporter but instead induces subtle changes in its conformational dynamics, resulting in altered protein flexibility, structural expansion, membrane interactions, and reorganization of the internal tunnel network. Within the nucleotide-free outward-facing conformational state represented by the cryo-EM structure used in this study, these structural rearrangements, together with the reshaping of the free energy landscape into more isolated conformational basins separated by a localized energy barrier, suggest that the arg230cys substitution reduces long-range allosteric communication and limits the conformational transitions required for efficient lipid transport. In particular, the redistribution of the internal tunnel network into multiple alternative pathways, combined with the increased conformational heterogeneity observed in the extracellular domains, may compromise the stability and persistence of a preferential cholesterol transport route toward ApoA-I. Collectively, these findings provide a plausible molecular explanation for the reduced cholesterol efflux and lower plasma HDL-C levels previously associated with the R230C variant, supporting the concept that subtle perturbations in protein dynamics, protein–lipid interactions, and conformational energetics can have significant functional consequences. More broadly, this study highlights the value of integrating molecular dynamics simulations with membrane biophysics to elucidate how naturally occurring missense variants reshape the conformational landscape of membrane transporters and contribute to transporter dysfunction and metabolic disease.

## Data Availability

The data generated and analyzed during this study are available within the article and its Supplementary Materials. Additional information is available from the corresponding author upon reasonable request.

## Supplementary Materials

The following supporting information can be downloaded at: https://www.mdpi.com/article/10.3390/ijms27188240/s1. The Supplementary Material provides further support for the findings presented in the main manuscript; including additional structural and conformational analyses of ABCA1 WT and the R230C variant, as well as complementary membrane analyses.

## Abbreviations

The following abbreviations are used in this manuscript:

ABCA1	ATP-binding cassette transporter A1
R230C	Arginine230Cysteine
WT	Wild type
ECD1	Extracellular domain 1
ECD2	Extracellular domain 2
NBD1	Nucleotide-binding domain 1
NBD2	Nucleotide-binding domain 2
TMD1	Transmembrane domain 1
TMD2	Transmembrane domain 2
RMSD	Root Mean Square Deviation
RMSF	Root Mean Square Fluctuation
Rg	Radius of gyration
RCT	Reverse Cholesterol Transport
SASA	Solvent Accessible Surface Area
K	Kelvin
JAK2	Janus quinasa 2
HDL	High density lipoprotein
HDL-C	Cholesterol High density lipoprotein
Arg	Arginine
Cys	Cysteine
K^+^	Potassium ion
Cl^−^	Chloride ion
POPC	1-palmitoyl-2-oleoyl-sn-glycero-3-phosphocholine
PSM	Palmitoylsphingomyelin
POPI	Palmitoyloleoyl phosphoinositol
POPE	1-palmitoyl-2-oleoyl-sn-glycero-3-phosphoethanolamine
POPS	1-palmitoyl-2-oleoyl-sn-glycero-3-phospho-L-serine
LPC	Lysophosphatidylcholine
NTP	Normal Temperature and Pressure
C75	Cys75
C309	Cys309
R	Arginine
ApoA-I	Apolipoprotein A-I
dPCA	Dihedral principal component analysis
dPCA	Principal component analysis
N	Nitrogen
PC1	Polycystin-1
PC2	Polycystin-2
MDS	Molecular Dynamics Simulations
ns	Nanosecond
nm	nanometers
