# Supplementary material for: Molecular Dynamics Simulations Reveal Structural Changes Associated with the ABCA1 R230C Functional Variant
Source: Int J Mol Sci. 2026 Sep 16;27(18):8240. doi: 10.3390/ijms27188240 (PMC13607428; doi:10.3390/ijms27188240)
Supplement: Supplementary file 1 [file ijms-27-08240-s001.zip › SupplementaryFiles/Supplementary Material.pdf]

Molecular Dynamics Simulations Reveal Structural Changes Associated with the *ABCA1* R230C Functional Variant

Supplementary material

Juan José de la Cruz-López[1], Luis Ramón Tercero Martínez-González[1,2], Cecilia Albortante-Morato[3], Rosaura Palma-Orozco[3], María Teresa Flores-Dorantes[1,*], Jorge Luis Rosas-Trigueros[3,*]

1. Laboratorio de Biología Molecular y Farmacogenómica, Centro de Investigación de Ciencia y Tecnología Aplicada de Tabasco (CICTAT), División Académica de Ciencias Básicas, Universidad Juárez Autónoma de Tabasco, Tabasco, México.
2. División Académica de Ciencias y Tecnologías de la Información, Centro de Investigación de Ciencia y Tecnología Aplicada de Tabasco (CICTAT), Universidad Juárez Autónoma de Tabasco, Tabasco, México.
3. Laboratorio Transdisciplinario de Investigación en Sistemas Evolutivos, Escuela Superior de Cómputo (ESCOM), Instituto Politécnico Nacional, Ciudad de México, México.

*Correspondence: jlrosas@ipn.mx (J.L.R.-T.) and mteresafd@gmail.com (M.T.F.-D.)

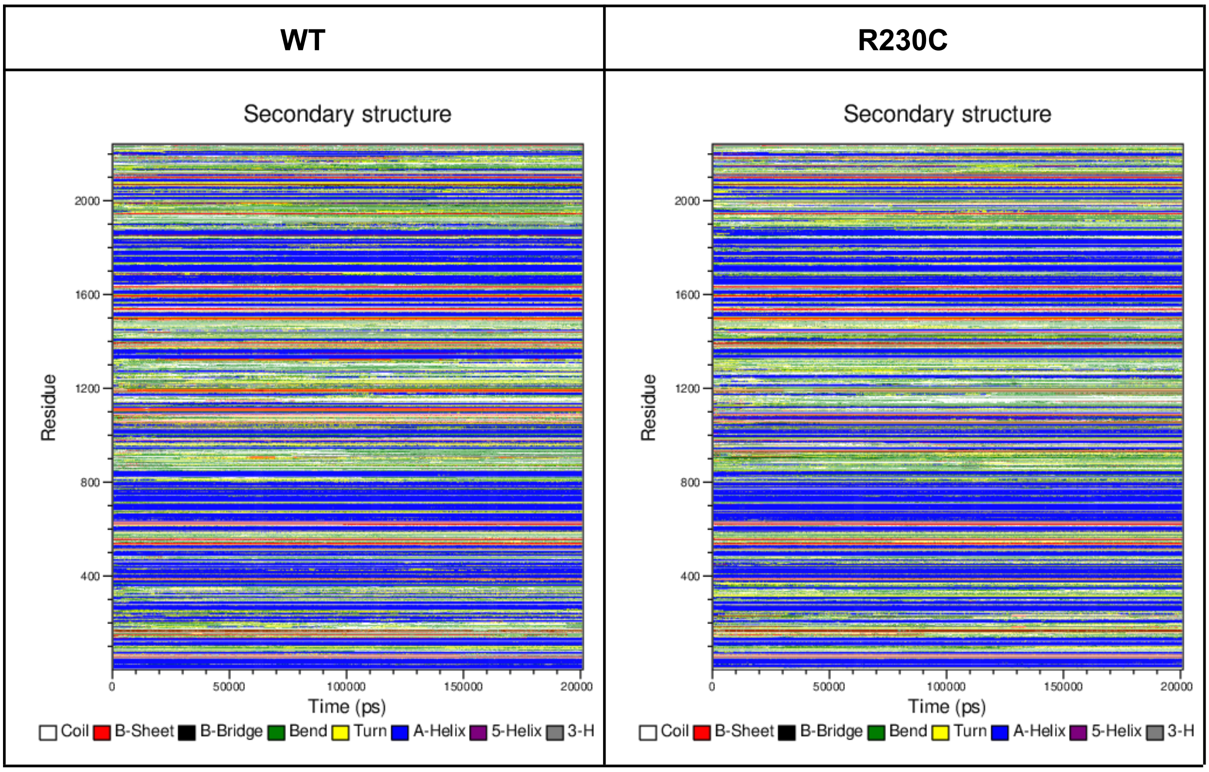

Supplementary Figure S1. Time-resolved secondary structure evolution of the full-length ABCA1 transporter during the 200 ns molecular dynamics simulations. Secondary structure assignments for the wild-type (WT, left) and R230C variant (right) were obtained using the DSSP algorithm implemented in GROMACS. Each horizontal line represents an individual residue, while the x-axis corresponds to simulation time. Colors indicate the assigned secondary structure states, including α-helix, 3_{10}-helix, π-helix, β-sheet, β-bridge, bend, turn, and coil. These residue-level maps complement the domain-based secondary structure comparison presented in **Figure 3**, providing a detailed visualization of the temporal stability and local secondary structure transitions throughout the simulations.

Supplementary Table S1. Summary of the total number of residues assigned to each major secondary structure category in wild-type (WT) ABCA1 and the R230C variant during the molecular dynamics simulation, as determined by DSSP analysis. The table reports the cumulative number of residues classified as α-helices, β-strands, turns, and coils, together with the total number of assigned residues for each protein. These residue-level assignments served as the basis for calculating the specific secondary structure percentages.

**Secondary structure**	**WT (residues)**	**R230C (residues)**
Helices	920	917
Strands	144	139
Turns	326	314
Coils	446	472
**Total assigned**	**1836**	**1842**

Supplementary Table S2. Residues identified as dynamic by DSSP-based secondary structure analysis in each structural domain of wild-type (WT) ABCA1 and the R230C variant throughout the MD simulation. Dynamic residues are listed according to their corresponding protein domain (ECD1, ECD2, TMD1, TMD2, NBD1, NBD2, RD1, and RD2). These residue-level assignments served as the basis for calculating the domain-wise percentages of dynamic residues shown in **Figure 4**.

**Domain**	**WT Dynamic Residues**	**R230C Dynamic Residues**
**ECD1**	59, 60, 75, 76, 77, 78, 89, 92, 93, 131, 132, 133, 134, 135, 136, 138, 139, 140, 141, 143, 148, 154, 157, 161, 165, 178, 179, 180, 181, 182, 183, 185, 186, 187, 192, 193, 194, 195, 196, 197, 198, 199, 200, 201, 210, 211, 213, 214, 215, 226, 227, 232, 241, 242, 243, 245, 246, 247, 248, 251, 252, 253, 254, 274, 290, 293, 294, 299, 300, 301, 302, 309, 310, 313, 316, 318, 323, 324, 325, 326, 327, 328, 329, 332, 333, 334, 335, 337, 339, 343, 344, 345, 347, 348, 349, 351, 353, 354, 387, 405, 406, 407, 420, 421, 429, 440, 454, 455, 456, 457, 471, 472, 480, 493, 494, 495, 529, 540, 541, 545, 560, 561, 562, 566, 567, 572, 573, 591, 592, 611, 615, 616	83, 87, 88, 89, 90, 91, 92, 94, 95, 96, 117, 118, 136, 137, 139, 140, 143, 144, 145, 146, 147, 152, 154, 155, 173, 174, 175, 177, 181, 184, 185, 186, 188, 189, 190, 191, 194, 195, 196, 197, 211, 212, 213, 217, 219, 220, 221, 222, 225, 226, 227, 228, 229, 230, 231, 232, 233, 234, 235, 236, 240, 243, 244, 245, 246, 247, 248, 275, 292, 293, 294, 295, 297, 299, 316, 317, 322, 323, 324, 325, 326, 330, 331, 332, 333, 334, 335, 336, 337, 338, 354, 355, 356, 357, 358, 360, 361, 363, 364, 387, 404, 407, 429, 431, 440, 451, 452, 453, 454, 455, 460, 461, 468, 469, 473, 477, 478, 481, 504, 529, 539, 543, 544, 545, 560, 561, 562, 566, 567, 570, 572, 584, 585, 586, 592, 615

**ECD2**	1371, 1372, 1373, 1380, 1382, 1395, 1397, 1398, 1399, 1400, 1401, 1419, 1420, 1421, 1422, 1424, 1425, 1426, 1431, 1447, 1448, 1449, 1450, 1451, 1452, 1453, 1457, 1459, 1462, 1469, 1470, 1471, 1475, 1480, 1487, 1523, 1542, 1599, 1600, 1620, 1621, 1624, 1625, 1626, 1638, 1639, 1640	1374, 1375, 1385, 1394, 1409, 1410, 1411, 1412, 1413, 1414, 1415, 1420, 1428, 1431, 1433, 1454, 1458, 1459, 1460, 1469, 1470, 1471, 1480, 1492, 1523, 1531, 1532, 1542, 1545, 1546, 1562, 1563, 1564, 1565, 1567, 1588, 1598, 1599, 1600, 1617, 1619, 1621, 1624, 1625, 1626, 1627, 1637
**TMD1**	20, 21, 648, 669, 670, 671, 672, 673, 674, 707, 741, 742, 767, 769, 770, 780, 781, 799, 802, 803, 805, 806, 807, 808, 809, 810, 816, 818, 819	648, 665, 674, 708, 741, 742, 772, 780, 781, 797, 798, 799, 800, 801, 805, 806, 810, 811, 815, 826, 828, 830
**TMD2**	1366, 1365, 1348, 1347, 1346, 1345, 1344, 1323, 1848, 1846, 1845, 1844, 1840, 1839, 1838, 1837, 1836, 1835, 1827, 1815, 1814, 1813, 1812, 1809, 1808, 1795, 1794, 1792, 1790, 1789, 1788, 1765, 1764, 1763, 1762, 1731, 1727, 1726, 1725, 1691, 1690, 1689, 1688, 1687, 1686, 1685, 1684, 1683, 1682, 1681, 1680, 1643, 1642, 1641, 1640, 1639	1651, 1665, 1666, 1678, 1680, 1683, 1684, 1685, 1686, 1687, 1692, 1699, 1725, 1726, 1728, 1734, 1750, 1751, 1752, 1758, 1759, 1762, 1784, 1788, 1789, 1792, 1795, 1796, 1797, 1798, 1809, 1838, 1839, 1847, 1848, 1871, 1872, 1873, 1874, 1342
**NBD1**	904, 908, 910, 912, 913, 914, 916, 918, 919, 926, 928, 929, 932, 935, 937, 939, 950, 951, 952, 953, 954, 959, 960, 962, 963, 964, 965, 966, 967, 986, 987, 988, 999, 1026, 1027, 1029, 1031, 1032, 1033, 1034, 1035, 1036, 1037, 1045, 1046, 1047, 1050, 1052, 1064, 1065, 1066, 1069, 1071, 1072, 1073, 1074, 1091, 1121, 1123, 1124, 1137, 1138, 1139, 1140, 1141	904, 905, 906, 908, 909, 910, 911, 912, 913, 914, 916, 917, 923, 924, 925, 936, 938, 947, 964, 965, 966, 967, 1000, 1001, 1015, 1026, 1027, 1031, 1032, 1033, 1035, 1036, 1037, 1060, 1061, 1062, 1066, 1067, 1068, 1069, 1070, 1071, 1079, 1096, 1100, 1101, 1105, 1106, 1107, 1111, 1112, 1113, 1114, 1122, 1132, 1141, 1142, 1144
**NBD2**	1907, 1908, 1910, 1911, 1916, 1917, 1928, 1929, 1931, 1932, 1933, 1934, 1935, 1939, 1945, 1947, 1948, 1949, 1950, 1951, 1952, 1953, 1954, 1955, 1956, 1957, 1958, 1967, 1972, 1974, 1977, 1978, 1979, 1980, 1987, 1988, 1996, 1997, 1999, 2000, 2015, 2017, 2018, 2032, 2035, 2036, 2037, 2038, 2039, 2040, 2046, 2047, 2048, 2058, 2059, 2060, 2062, 2079, 2093, 2101,	1907, 1908, 1909, 1910, 1911, 1912, 1913, 1914, 1915, 1928, 1929, 1930, 1931, 1938, 1939, 1949, 1958, 1967, 1970, 1975, 1979, 1980, 1984, 1985, 1986, 1996, 1999, 2000, 2017, 2035, 2036, 2040, 2044, 2057, 2058, 2059, 2071, 2072, 2093, 2116, 2117, 2118, 2119, 2120, 2123, 2124, 2125, 2126, 2131, 2132, 2133, 2134, 2137, 2140, 2141

	2109, 2110, 2121, 2128, 2129, 2130, 2135, 2136, 2137, 2138	
**RD1**	*(Not analyzed)*	*(Not analyzed)*
**RD2**	*(Not analyzed)*	*(Not analyzed)*

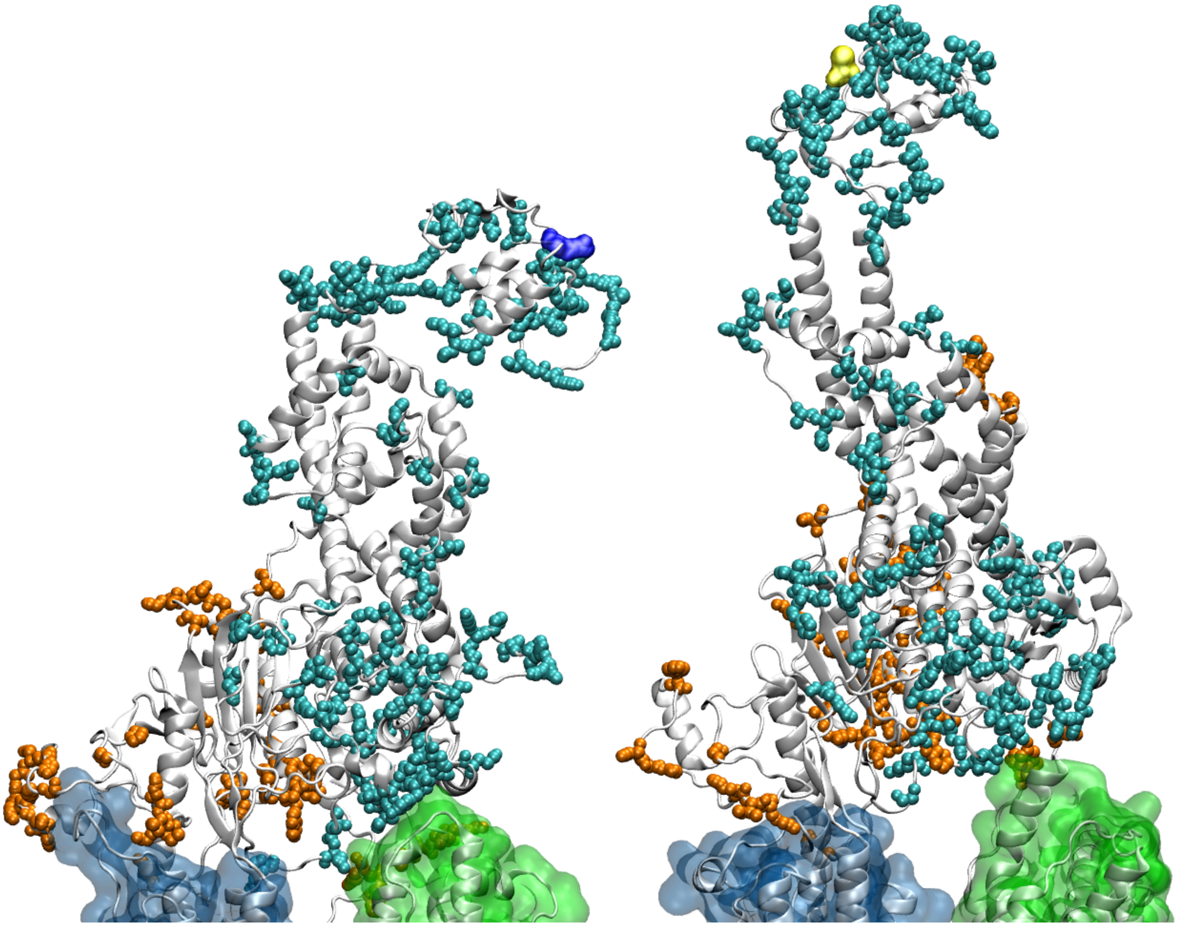

Supplementary Figure S2. Dynamic Residues. Average structures obtained from dPCA cluster analysis for WT (left), where R230 is depicted with a blue surface and R230C (right), where C230 is depicted with a yellow surface. Dynamic residues in ECD1 are depicted as cyan spheres, dynamic residues in ECD2 are depicted as orange spheres.

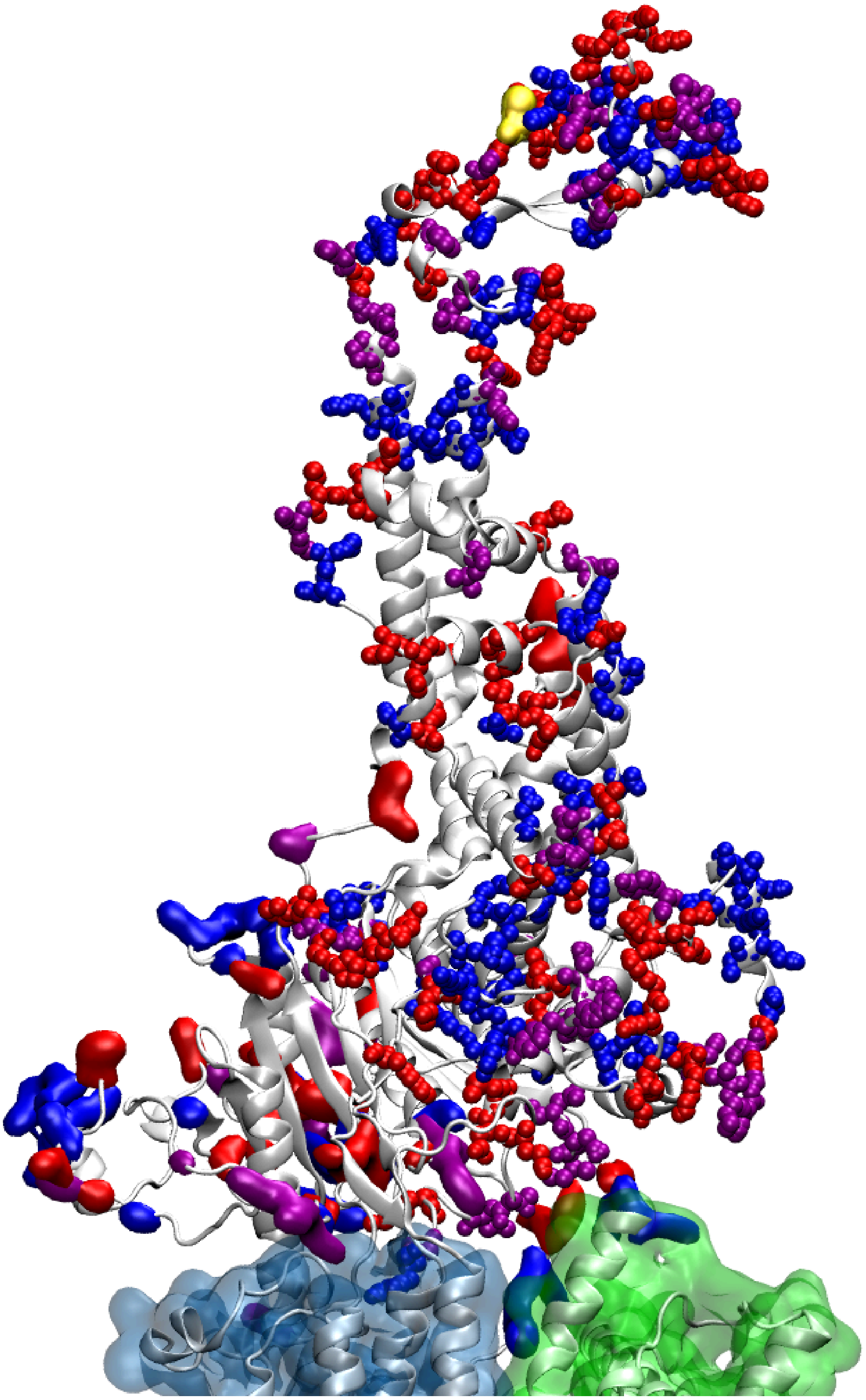

Supplementary Figure S3. Dynamic Residues intersection. Average structure for R230C. Residues that become dynamic in both WT and R230C are in purple. Residues that are found to be dynamic only in WT are in red, only in R230C are in blue. Residues in ECD1 are in spheres, residues in ECD2 are in surfaces.

A)

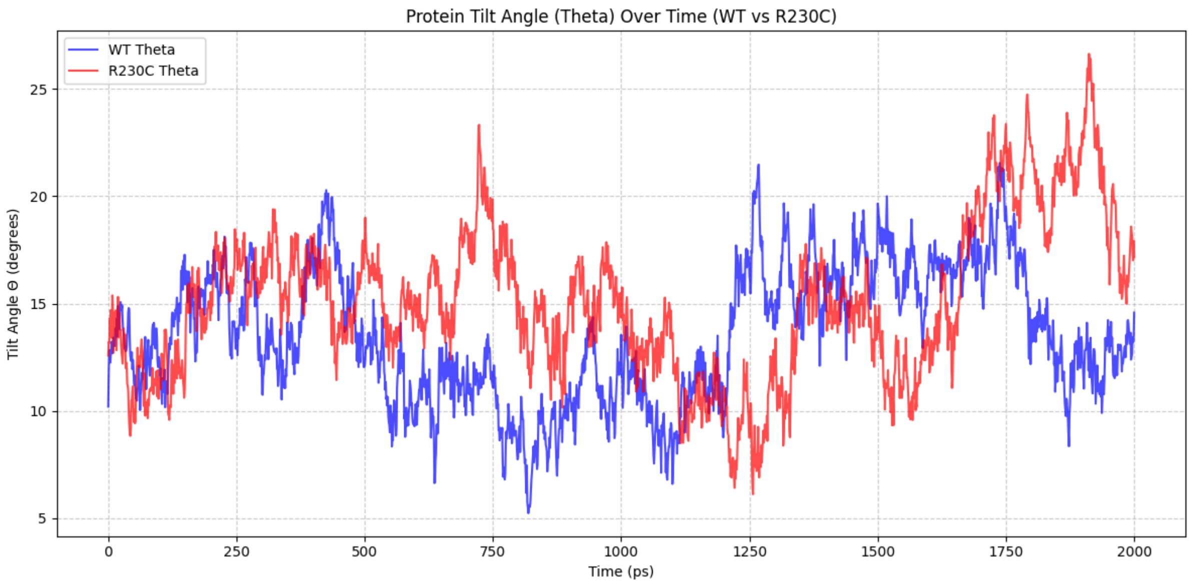

B)

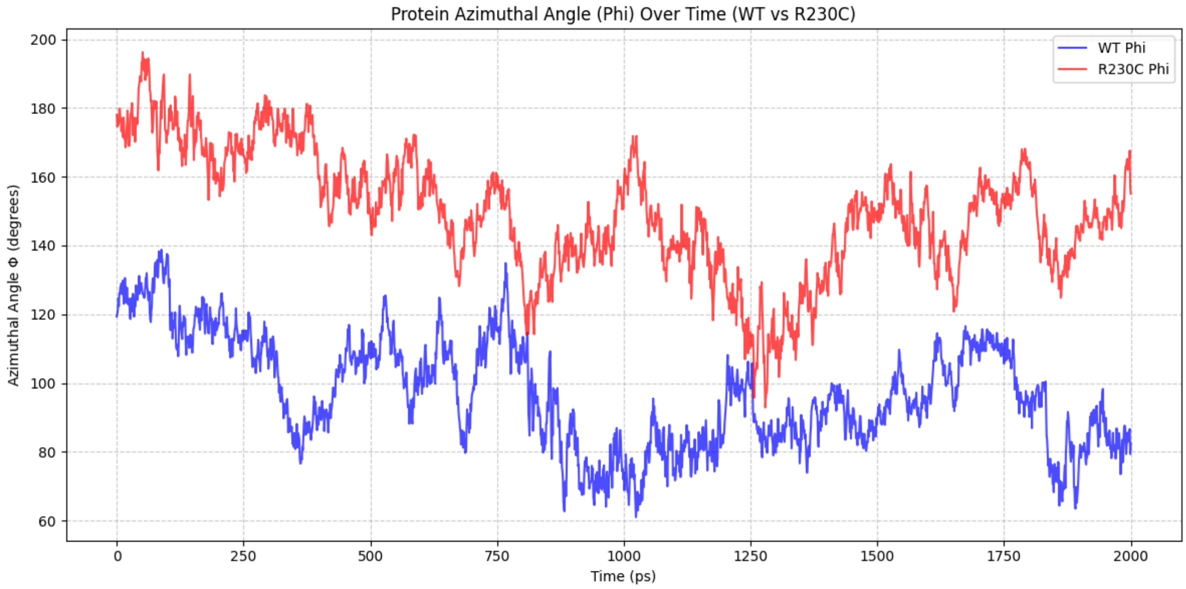

Supplementary Figure S4. Time evolution of ABCA1 orientation within the membrane during molecular dynamics simulations. (A) Protein tilt angle (Θ) and (B) azimuthal angle (Φ) of wild-type (WT, blue) and R230C (red) ABCA1 calculated throughout the simulations. These analyses complement the lipid orientation maps presented in Figure 6 by monitoring the temporal changes in protein orientation relative to the membrane. The trajectories illustrate the dynamic fluctuations of both orientation parameters for the WT and R230C systems over the simulation time.

A)

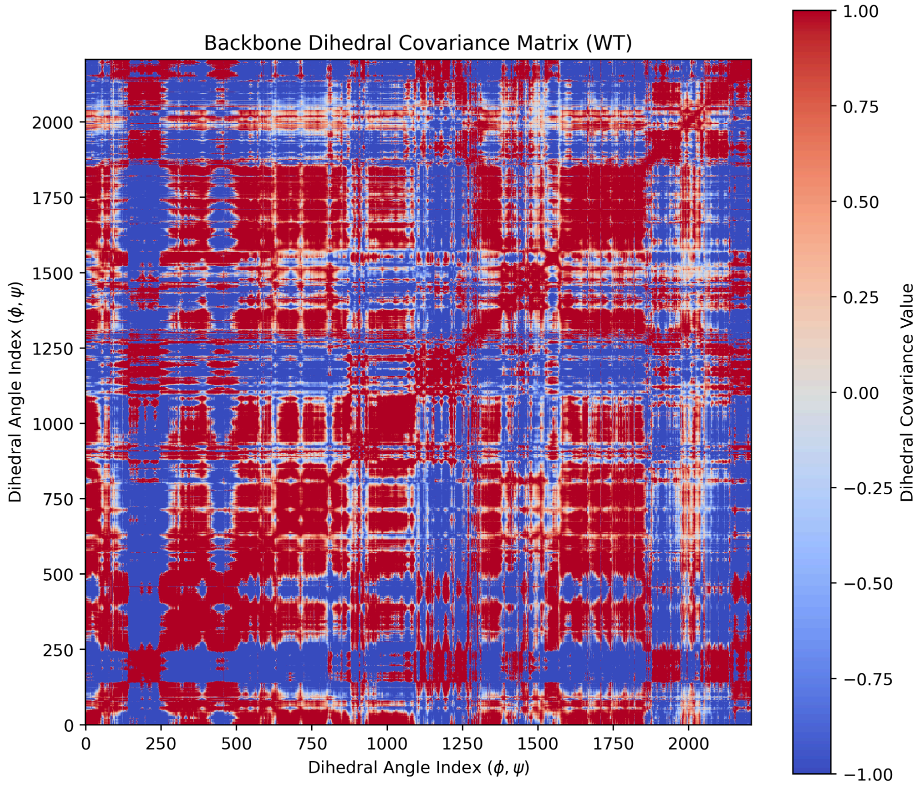

B)

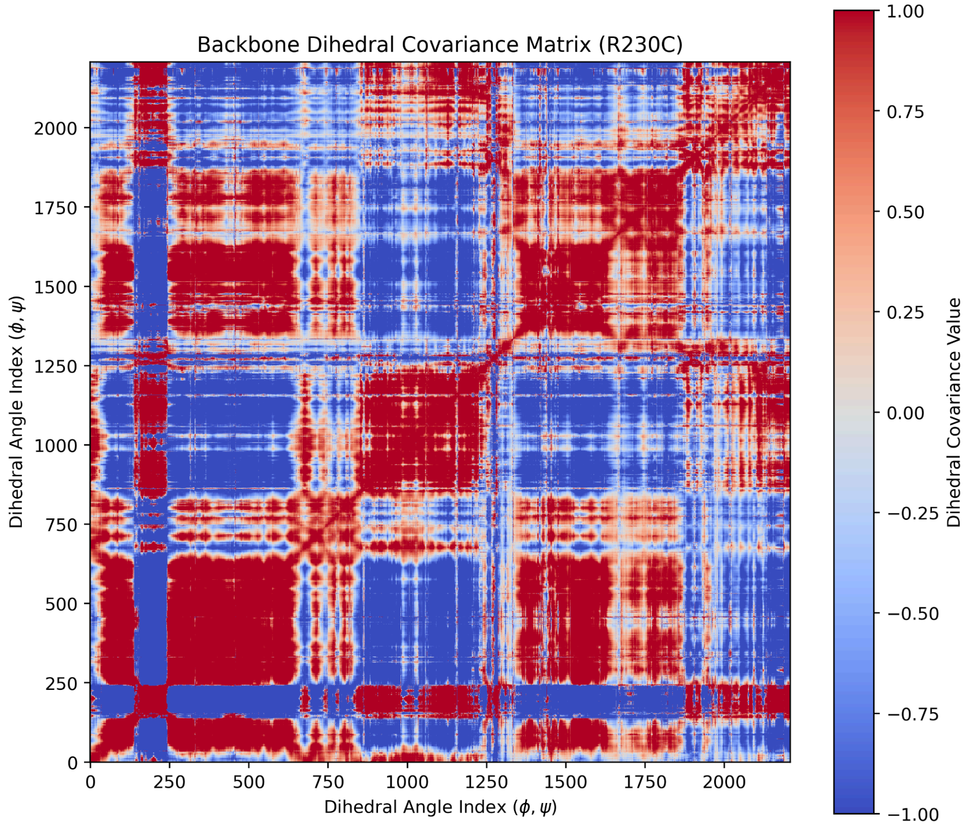

Supplementary Figure S5. 2D Backbone Dihedral Variance-Covariance Matrices for Human ABCA1. Full-matrix heatmaps constructed from dihedral principal component analysis (dPCA)

trajectories evaluating backbone (ϕ,ψ) internal coordinate covariance across all residues for **(A) Wild-Type (WT)** and **(B) R230C variant**. Color scales represent the magnitude of variance (diagonal) and long-range correlated/anti-correlated fluctuations (off-diagonal). The R230C variant exhibits a marked fragmentation and redistribution of off-diagonal covariance blocks relative to WT, demonstrating disrupted allosteric coupling between the extracellular domain 1 (ECD1) polymorphic site and distal catalytic domains (NBD2 and TMD2).

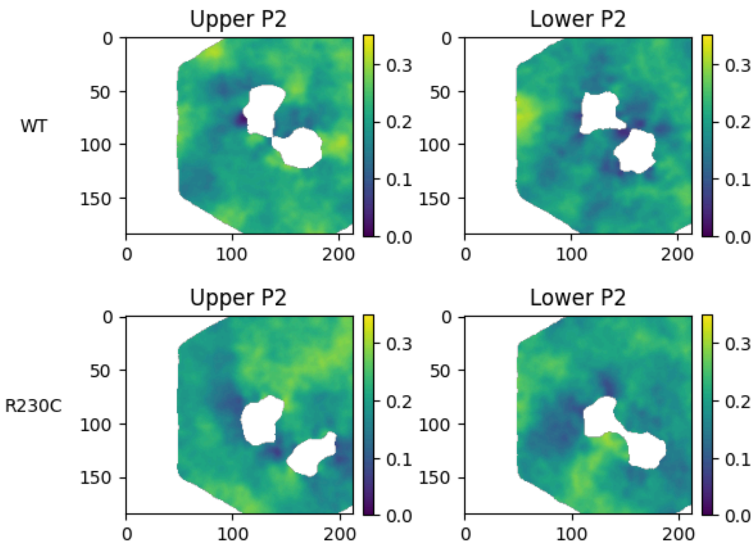

Supplementary Figure S6. Time-averaged spatial distribution of the lipid tail order parameter (P2) around WT ABCA1 and the R230C variant obtained from the molecular dynamics simulations. The upper (left) and lower (right) membrane leaflets are shown for each system. Colors represent the average rank-2 lipid tail order parameter (P2), ranging from low lipid ordering (purple) to high lipid ordering (yellow). White areas indicate the protein footprint, where lipid order parameters were not calculated.

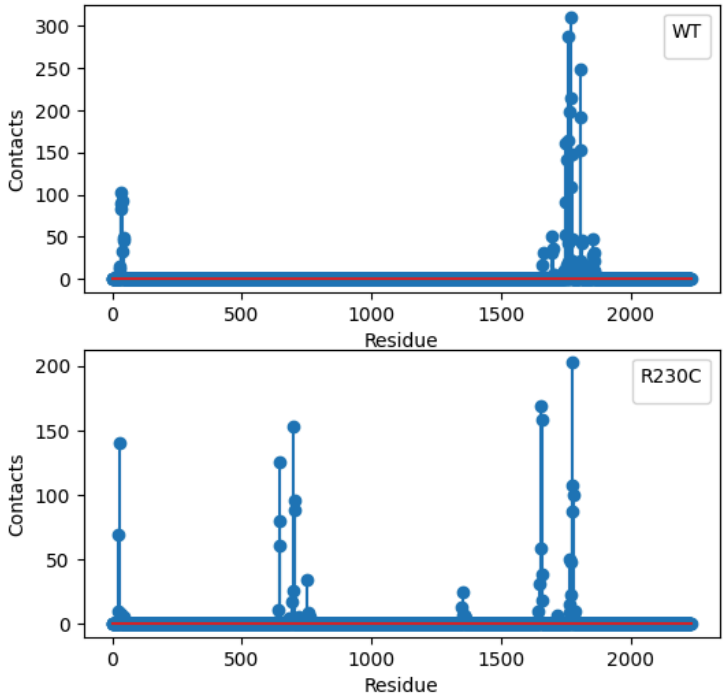

Supplementary Figure S7. Average number of contacts formed between individual ABCA1 residues and cholesterol molecules during the 200 ns molecular dynamics simulations. Contact frequencies are shown as a function of residue number for the WT (top) and R230C (bottom) systems. Peaks represent residues exhibiting the highest frequency of cholesterol interactions throughout the trajectories.

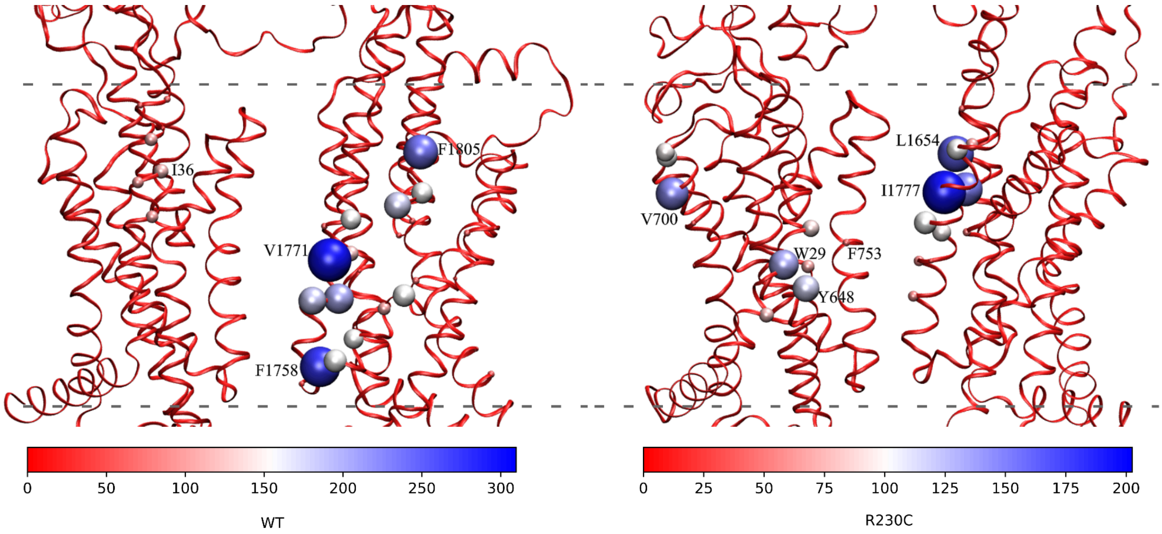

Supplementary Figure S8. Depiction of the average number of contacts between each protein residue and Cholesterol molecules. The radius of the C-alpha atom is proportional to the number of contacts. The number of contacts is also shown by color. All interactions take place in the transmembrane domain. Selected residues are labeled. The regions that show interactions for WT are TM1 (F32, L35, I36, S39), TM10 (L1750, M1751, A1754), the loop between TM10 and TM11 (V1757, F1758), TM11 (V1767, L1768, V1771, N1772, I1775) and EH3 (F1805, F1808, P1809). For R230C, the regions that show interactions are TM1 (L25,W29), TM2 (L644, I647, Y648), TM3 (V700, K703, L704), TM5 (F753), TM8 (V1653, L1654, I1657) and TM11 (V1767, S1770, L1773, F1774, I1777, V1781).

Supplementary Table S3. Quantitative summary of the tunnel clusters identified by CAVER in the wild-type (WT) ABCA1 during the molecular dynamics simulation. Summary information about all identified tunnel clusters for WT. These quantitative data complement the representative tunnel visualization shown in Figure 7A.

ID	No	No_snaps	Avg_BR	SD	Max_BR	Avg_L	SD	Avg_C	SD	Priority	Avg_throughput	SD
1	20	20	1.695	0.346	2.65	19.822	6.199	1.351	0.13	0.69227	0.69227	0.08401
2	20	20	1.488	0.298	2.27	14.644	7.411	1.332	0.265	0.66832	0.66832	0.11457
3	20	20	1.431	0.405	2.67	41.982	24.69	1.696	0.562	0.46061	0.46061	0.20155
4	19	19	1.281	0.198	1.74	40.686	21.05	2.25	0.675	0.38139	0.40146	0.17646
5	20	20	1.24	0.231	2.02	56.736	27.35	2.088	0.563	0.31269	0.31269	0.20524
6	19	19	1.251	0.17	1.65	49.805	17.176	2.523	0.685	0.30535	0.32142	0.10794
7	17	17	1.2	0.261	2.13	60.011	30.369	2.174	0.594	0.2402	0.28259	0.17105
8	12	12	1.113	0.113	1.36	82.82	26.441	2.284	0.462	0.10092	0.16819	0.1276
9	10	10	1.063	0.052	1.15	97.317	29.374	2.121	0.202	0.0515	0.10301	0.05328
10	2	2	1.097	0.085	1.18	106.123	8.13	2.56	0.509	0.00754	0.07543	0.00734

Supplementary Table S4. Quantitative summary of the tunnel clusters identified by CAVER in the ABCA1 R230C variant during the molecular dynamics simulation. Summary information about all identified tunnel clusters for WT. These quantitative data complement the representative tunnel visualization shown in Figure 7B.

ID	No	No_snaps	Avg_BR	SD	Max_BR	Avg_L	SD	Avg_C	SD	Priority	Avg_throughput	SD
1	17	17	1.587	0.516	3.06	22.316	15.222	1.469	0.347	0.55575	0.65383	0.20792
2	15	15	1.512	0.584	2.82	23.073	32.161	1.879	1.221	0.49025	0.65367	0.25357
3	17	17	1.26	0.249	1.81	43.232	20.196	1.952	0.459	0.36433	0.42862	0.16738
4	17	17	1.293	0.301	2.02	45.784	23.503	1.83	0.425	0.34348	0.40409	0.19209
5	10	10	1.404	0.496	2.62	25.828	8.868	1.754	0.448	0.25383	0.50765	0.16827
6	17	17	1.185	0.195	1.78	71.73	25.667	2.47	0.462	0.21032	0.24744	0.15929
7	15	15	1.255	0.313	2.02	70.092	33.42	2.298	0.658	0.19027	0.25369	0.15398
8	17	17	1.224	0.226	1.79	82.859	37.358	2.143	0.411	0.18564	0.2184	0.15251
9	16	16	1.163	0.198	1.78	84.337	30.285	2.224	0.553	0.14526	0.18158	0.18189
10	8	8	1.36	0.527	2.69	73.339	33.401	1.893	0.444	0.11255	0.28138	0.26445
11	11	11	1.167	0.23	1.78	82.305	33.286	2.51	0.617	0.1119	0.20346	0.15936
12	9	9	1.102	0.118	1.34	84.202	53.367	2.058	0.551	0.10145	0.22545	0.19338
13	10	10	1.127	0.108	1.29	101.863	14.403	2.559	0.378	0.05987	0.11974	0.04728
14	8	8	1.161	0.191	1.64	103.893	17.782	2.359	0.423	0.05105	0.12763	0.08227
15	6	6	1.154	0.092	1.34	134.897	34.321	2.573	0.408	0.02304	0.07679	0.03419
16	6	6	1.151	0.103	1.26	173.705	30.594	2.411	0.296	0.01099	0.03663	0.01812
17	1	1	1.077	0	1.08	183.313	0	1.961	0	0.00118	0.02363	0

```
           ID: Identification of a given tunnel cluster = ranking
of a given cluster
                based on the Priority.

           ID: Identification of a given tunnel cluster = ranking
of a given cluster
                based on the Priority.
            No: Total number of tunnels belonging to a given
cluster.
      No_snaps: Number of snapshots with at least one tunnel
                with a radius >= probe_radius (see config.txt).
        Avg_BR: Average bottleneck radius [A].
        Max_BR: Maximimum bottleneck radius [A].
         Avg_L: Average tunnel length [A].
         Avg_C: Average tunnel curvature.
      Priority: Tunnel priority calculated by averaging tunnel
                throughputs over all snapshots (zero value used
for
                snapshots without tunnel).
Avg_throughput: Average tunnel throughput.
   Avg_up_E_BR: Average upper error bound of bottleneck radius
estimation.
   Avg_up_E_TR: Average upper error bound of tunnel profile radii
estimation.
   Max_up_E_BR: Maximal upper errors bound of bottleneck radii
estimation.
   Max_up_E_TR: Maximal upper error bound of tunnel radii
estimation.
            SD: Standard deviation [A].
```

All averaged values are calculated over snapshots, where a given cluster has at least one tunnel. The only exception is the Priority, where the average is calculated over all snapshots. If there are more tunnels in one snapshot, the cheapest tunnel is chosen.
